# Dietary disodium fumarate supplementation alleviates subacute ruminal acidosis (SARA)-induced liver damage by inhibiting pyroptosis *via* mitophagy-NLRP3 inflammasome pathway in lactating Hu sheep

**DOI:** 10.3389/fimmu.2023.1197133

**Published:** 2023-05-19

**Authors:** Hongzhu Zhang, Huimin Shi, Shendong Zhou, Guozhen Wei, Wan Xie, Meijuan Meng, Guangjun Chang, Xiangzhen Shen

**Affiliations:** Ministry of Education Joint International Research Laboratory of Animal Health and Safety, College of Veterinary Medicine, Nanjing Agricultural University, Nanjing, Jiangsu, China

**Keywords:** SARA, liver damage, NLRP3 inflammasome, pyroptosis, mitophagy

## Abstract

Liver damage is common in ruminants with subacute ruminal acidosis (SARA). Disodium fumarate (DF) could regulate rumen microbial community and neutralize ruminal organic acids. This study aimed to evaluate the effect of dietary DF supplementation on SARA-induced liver damage and investigate the underlying mechanism. The results showed that feeding a high-concentrate diet induced decreased rumen fluid pH and increased ruminal LPS. The rumen fluid pH in the HC group was less than 5.6 at 4 time points, indicating that SARA was successfully induced. The histopathological analysis showed that in the HC group, hemorrhage and inflammatory cell infiltration were observed in liver tissue. Using ELISA kits and biochemical analyzer, we identified that the contents of interleukin 1beta (IL-1β), interleukin 18 (IL-18), caspase-1, and the activity of alanine aminotransferase (ALT) and aspartate aminotransferase (AST) in hepatic vein were elevated in the HC group. However, DF supplementation increased rumen fluid pH value, decreased ruminal LPS, attenuated hemorrhage and inflammatory cell infiltration in the liver tissue, and decreased contents of IL-1β, IL-18, caspase-1, AST, and ALT in the hepatic vein. Real-time PCR and western blot analysis displayed that SARA-induced increased expression of pyroptosis-related proteins (GSDMD-NT) was attenuated in the HCDF group. Meanwhile, SARA induced increased expression of mitophagy and inflammasome-related proteins (MAP1LC3-II, PINK1, Parkin, cleaved-caspase-11, cleaved-caspase-1, NLRP3, and ASC) and elevated expression of inflammasome-related genes (NLRP3, CASP1, and ASC), which was reversed by DF supplementation. Moreover, SARA activated toll-like receptor 4 (TLR4)-nuclear factor kappa B (NF-κB) signaling pathway and inhibited the entry of forkhead box A2 (FOXA2) into the nucleus, which was reversed by DF supplementation. Collectively, our data suggest that dietary DF supplementation inhibited hepatocyte pyroptosis by regulating the mitophagy-NLRP3 inflammasome pathway and the NF-κB signaling pathway, thus alleviating SARA-induced liver damage in Hu sheep.

## Introduction

Feeding a high-concentrate diet is widely applied in postpartum ruminants because it brings improved production performance. However, long-term feeding of high-concentrate diets will lead to subacute ruminal acidosis (SARA). SARA is a common digestive disease characterized by a persistent low rumen pH below 5.6 for 3 h after feeding per day (1). The mechanism of SARA has been elucidated in detail in previous studies. Generally, feeding a high-concentrate diet increases rumen carbohydrate content, and elevates the production of volatile fatty acid (VFA) and lactic acid through microbial fermentation, thus decreasing rumen pH (2–4). SARA could induce milk fat depression, causing reduced milk quality. A previous survey showed that SARA reduced milk protein content by 0.12%, milk fat content by 0.3%, and milk yield by 2.7 kg/d (2). In addition, SARA can trigger a variety of metabolic diseases such as liver abscess, diarrhea, and laminitis (2). The liver collects blood from the rumen and delivers it to the posterior vena cava. When SARA occurs, because of low rumen pH, gram-negative bacteria lysate and release large amounts of lipopolysaccharide (LPS) into the rumen. Free LPS flows into the portal vein through damaged rumen epithelium caused by low rumen pH and reaches the liver, provoking liver damage (3, 5). Our previous study revealed that the liver exerted a role in LPS clearance by measuring the LPS concentrations in the portal vein and hepatic vein, which indicated that the liver plays an important role in preventing SARA-induced metabolic disease (6). Therefore, investigating the mechanism of SARA-induced liver damage and finding a potential treatment for SARA-induced liver damage is necessary.

Pyroptosis is a programmed necrotic cell death, which is first reported by Cookson et al. in 2001 (7). It is characterized by the formation of cell membrane pores and the release of mature interleukin 1beta (IL-1β) and interleukin 18 (IL-18) (7, 8). Pyroptosis is closely related to the activation of inflammasome. NLR family pyrin domain-containing 3 (NLRP3) inflammasome is the most well-known inflammasome family protein. There are two pathways of NLRP3 inflammasome activation depending on the ligands: one is the canonical pathway driven by pathogen-associated molecular patterns (PAMPs) and damage-associated molecular patterns (DAMPs) and mediated by caspase-1, another is non-canonical pathway driven by LPS and mediated by caspase-11 (caspase-4/5 in humans) (9). It has been widely demonstrated that LPS is an important initiator for the caspase-4/5/11-dependent activation of NLRP3 inflammasome. LPS could directly activate caspase-11 independent of toll-like receptor 4 (TLR4) (9). Then, the activated caspase-11 causes the cleavage of gasdermin D (GSDMD) into GSDMD-N terminal (GSDMD-NT) and GSDMD-C terminal (GSDMD-CT). The GSDMD-NT forms oligomers and attaches to the cell membrane to form pores (8). Meanwhile, GSDMD-NT promotes NLRP3, apoptosis-associated speck-like protein containing a card (ASC) and pro-caspase-1 to form NLPR3 inflammasome complex, activating caspase-1 (9). The activated caspase-1 promotes the cleavage of pro-IL-1β and pro-IL-18 into mature IL-1β and IL-18, which flow out of the cell through membrane pores and cause an inflammatory response and tissue damage (10).

Mitophagy is a selective autophagy characterized by the specific phagocytosis of damaged mitochondria (11), and plays an important role in intracellular quality control, inflammation, apoptosis, and many other biological processes (12, 13). Mitochondria is the main site for the production of cellular reactive oxygen species (ROS), and mitochondria ROS (mtROS) has been reported to engage in the activation of NLRP3 inflammasome (14, 15). It has been widely demonstrated that mitophagy exerts a cytoprotective role *via* eliminating mtROS to inhibit the activation of NLRP3 inflammasome, leading to ameliorated pyroptosis (16, 17). However, the study on pyroptosis and mitophagy in ruminants with SARA has not been reported.

Fumarate is a metabolic intermediate of the tricarboxylic acid cycle, and disodium fumarate (DF) is often used as a feed additive to improve ruminal fermentation (18). Accumulating evidence has revealed that dietary DF supplementation can increase rumen pH. Dietary DF supplementation could increase rumen pH in sheep fed a high-forage diet (18). Jin et al. reported that DF supplementation increased rumen pH and the concentration of lactate in an *in vitro* rumen fermentation experiment (19). Research on the rumen microbiota showed that dietary DF supplementation modified the ruminal microbial community and increased the number of lactic acid-utilizing bacteria, thus decreasing the ruminal lactic acid (19, 20). Moreover, as a strong alkali and weak acid salt, DF itself has a certain buffering capacity. However, whether dietary DF supplementation could be a potential therapeutic strategy for SARA-induced ruminant liver damage remains investigated.

Here, we hypothesize that SARA induces hepatocyte pyroptosis and leads to liver damage in Hu sheep fed a high-concentrate diet. We also hypothesize that dietary DF supplementation could alleviate SARA-induced liver damage by regulating rumen pH and attenuating hepatocyte pyroptosis. To validate these, animals were randomly divided into 3 groups and fed a low-concentrate diet, a high-concentrate diet, and a high-concentrate diet with DF supplementation for 8 wk to investigate the effect of DF supplementation on the mitophagy, NLRP3 inflammasome activation, and pyroptosis in the livers of lactating Hu sheep.

## Materials and methods

### Ethics statement

The animal experiments were examined and approved by the Animal Care and Use Committee of Nanjing Agricultural University (NJAU.No20220112003), and all animal operations strictly complied with the experimental protocols under the Ministry of Science and Technology’s Law on Experimental Animals (2006, Beijing, China).

### Animals, diet, and experimental design

Eighteen healthy lactating Hu sheep (parities 2-3, 49.8 ± 5.0 kg BW) were installed with rumen fistula followed by a 2-wk adaption period, during which all sheep were fed the LC diet to adapt to the environment. After then, all sheep were randomly divided into a low-concentrate group (LC group, forage: concentrate=7:3, n=6), a high-concentrate group (HC group, forage: concentrate=3:7, n=6), and a high-concentrate diet with disodium fumarate supplementation group (HCDF group, forage: concentrate=3:7, 10 g DF per sheep daily, n=6). The experiment lasted for 8 wk. Animals were fed at 0800 and 1700 h per day. The specific ingredients in the diet are listed in **Table 1**. During the experimental period, animals were housed in individual tie stalls and free to access water, and their body was monitored daily by checking rectal temperature, respiratory rate and feed intake. All animals were healthy during the experimental period.

**Table 1:** The ingredients and nutrient composition of treatment diets.

Item	Diets^1^		
	LC	HC	HCDF
Ingredients (% of dry matter)			
Ground corn	19.28	46.86	46.86
Soybean meal	4.20	9.80	9.80
Bran	2.70	8.10	8.10
Rapeseed meal	1.20	2.80	2.80
Limestone	0.42	0.82	0.82
Salt	0.50	0.50	0.50
Calcium hydrophosphate	1.20	0.62	0.62
Premix^2^	0.50	0.50	0.50
Peanut vine	35.00	15.00	15.00
Silage corn	35.00	15.00	15.00
Disodium fumarate (g/sheep/d)	0	0	10.00
Nutritional composition			
Crude protein (%)	9.75	13.53	13.53
Crude fiber (%)	6.70	5.15	5.15
Ca (%)	0.60	0.60	0.60
P (%)	0.40	0.40	0.40
Neutral detergent fiber (%)	40.66	24.41	24.41
Acid detergent fiber (%)	20.56	12.14	12.14
Ash (%)	3.54	2.98	2.98
Digestible energy (KJ/kg)	9.09	11.64	11.64

^1^LC = low-concentrate diet; HC = high-concentrate diet.

^2^Premix ingredient: VA, 4 kIU/kg; VD_3_, 400 IU/kg; VE, 20 kIU/kg; FeSO_4_, 69.03 mg/kg; CuSO_4_ 17.6 mg/kg; K_2_SO_4_, 31.7 mg/kg; ZnSO_4_, 57.14 mg/kg; MnSO_4_, 44.03 mg/kg; CoCl_2_, 0.25 mg/kg; Na_2_SeO_3_, 8.95 mg/kg; NaHCO_3_, 740.91 mg/kg; Monensin, 6.00mg/kg.

### Sample collection

On d 7, 10, 14, 18, 21, 28, 35, 42, 48, 54, 55, and 56 of the experiment period, ruminal fluid was collected from the ventral sac of the rumen through the rumen fistula before feeding in the morning, which was 0 h, and then was collected at 1, 2, 3, 4, 5, 6, and 8 h after feeding. After filtering the rumen fluid with a 4-layer of gauze, the rumen fluid pH value was assessed with a pH meter (HI 9125, Hanna Instruments, Italy). At the end of the experiment, each sheep was anesthetized with 0.5 mL of xylazine hydrochloride. After collecting blood samples, all sheep were slaughtered, and liver tissue was collected and divided into small pieces. Some of the liver samples were stored in liquid nitrogen and the others were stored in 4% paraformaldehyde.

### RNA extraction, cDNA synthesis, and real-time PCR

Total RNA was extracted using an RNA extraction kit (Cat # 9109) from TAKARA (Japan) according to the manufacturer’s instructions. Then, 500 ng of RNA, 2 μL of 5×All-in-one qRT SuperMix and 0.5 μL of Enzyme Mix were mixed, and adding nuclease-free water to a total of 10 μL. The reactions were carried out in a Mastercycler Nexus (Eppendorf).

The cDNA was diluted with nuclease-free water for subsequent experiments. 10 μL of 2×ChanQ Universal SYBR qPCR Master Mix (Cat # Q711, Vazyme, Nanjing, China), 5 μL of cDNA, 0.8 μL each of 10 μM forward and reverse primer, and 3.4 μL of nuclease-free water were added to a 200-μL Eppendorf tube. The reactions were conducted in an ABI 7300 Real-Time PCR system (Thermo Fisher, San Jose, CA, USA). The primers used in this experiment were designed by Oligo 7 software (Molecular Biology Insights, Inc.) and are listed in **Table 2**. There were no significant differences in the cycles-to-threshold values of actin beta (*ACTB*) among the three groups. Therefore, *ACTB* was used as an internal reference, and relative abundance of target genes was determined through the 2*^-△△Ct^* method.

**Table 2:** Primers used in quantitative real-time PCR analysis.

Target gene^1^	Forward (5´-3´)	Reverse (5´-3´)	Production length(bp)	Accession number
*ACTB*	GGCACCACACCTTCTACAACGA	ATCTTCTCACGGTTGGCCTT	100	NM_001009784.3
*BECN1*	GAATGCACAGACACCCT	ATTTTCTGCCACTATCTTCCG	219	XM_005693865.3
*ATG5*	GAAGTGCCAAATACAGTCCT	CCCCAGCTCTGAACACC	196	XM_005684610.3
*SQSTM1*	ACCCGTCTACAGGTGAACTCC	ATGCAGCTTCCTTCAGTCCT	105	XM_018051607.1
*GSDMD*	CTTCCTGTCCGGCTTGCT	AGTGCCGAGTCCTTAACCAAC	97	XM_018058675.1
*IL1B*	AATATGGAAAAGCGATTCGTCT	GCCACCTCTAAAACGTCCCA	141	XM_013967700.2
*IL18*	ATACGAAATTTGAACGACCA	AGCCAGACCTCTAGTGA	150	NM_001285544.1
*ASC*	CGTGGACCTTACCGACAAGC	TGGCACGTTTCTAGCACCCT	148	XM_005697733.3
*NLRP3*	GCTGCCATGTACTACCTGC	AATTTGCCGTAGTTTTCGAGA	119	XM_005682796.3
*CASP1*	CCAGACATTCAACAACCGTA	CTCTTCCAGGTTCTCGGAT	160	XM_018058933.1
*IL8*	AGTACAGAACTTCGATGCCAA	AGCACACCTCTTTTCCGTTG	148	XM_005681749.3
*NFKB1*	CCAGAGTTTACGCCTGA	AGACCTCGTAGTTGTCC	211	XM_018049264.1
*TLR4*	TCTCTACAAAATCCCCGAC	AATCTTAATTTCGCATCTGG	145	NM_001285574.1
*NFKBIA*	GGTGAAGGAGCTGCGAGAG	GCTCACAGGCAAGGTGTAGG	326	XM_018066509.1
*CAT*	ACTGTTTCCGTCCTTTATCCACA	ACCAGCTTGAAAGTATGCGAT	192	XM_004016396.5
*NQO1*	CCAAGTAGCCTCTTTGACC	ATGGCTTTGATCTGATTGTCC	146	XM_004015102.5
*GPX1*	CTTCCCGTGCAACCAGT	GCCTTCTCGCCATTCACCT	141	XM_004018462.5
*SOD1*	AAGGCACCATCCGCTTCGAG	TCCTTTGGCCCACCGTGTT	178	NM_001145185.2
*SOD2*	GCCTACGTGAACAACCTCA	AAAGGAACCAAAGTCACGTT	216	NM_001280703.1
*SOD3*	CTGCTCCTCGGGTCCCA	ATCTGCTCCTCCGTGTT	167	XM_015096418.3
*HMOX1*	CATGCCCCAGGATTTGTCAG	TTCTCCTTGTTGCGTTCGAT	189	XM_027967703.2
*HMOX2*	CAACGCACAGCAGTTCAAGC	AATACCTGCATGTTGAACTCG	123	XM_027962068.2
*GSTP1*	CCGCCCTACACCATCGTCT	CTCATCTGTGCCGTAGAGCC	249	XM_027959471.2

^1^ACTB, actin beta; ATG5, autophagy related 5; SQSTM1, sequestosome 1; GSDMD, gasdermin D; IL-1β, interleukin-1beta; IL-18, interleukin-18; ASC; apoptosis-associated speck-like protein containing a CARD; NLRP3, NLR family PYRIN domain-containing 3; Caspase-1, apoptosis-related cysteine protease; IL-8, interleukin-8; NFKB1, nuclear factor kappa B subunit 1; TLR4, toll-like receptor 4; NFKBIA, nuclear factor kappa B inhibitor alpha; CAT, Catalase; NQO1, NAD(P)H quinone dehydrogenase 1; GPX1, Glutathione peroxidase 1; SOD, Superoxide dismutase; HMOX, Heme oxygenase; GSTP1, Glutathione S-transferase pi 1.

### Western blot analysis

Western blot analysis was conducted as previously described (21). Briefly, grinding liver samples into powder with liquid nitrogen. Then, 100 mg of tissue powder, 1 mL of protein lysate, 10 μL of protease inhibitor, and 10 μL of protein phosphatase inhibitor were added to a 1.5-mL Eppendorf tube. After mixing well for 10 min, protein supernatant was isolated through centrifugation (15984 × g, 4°C, 15 min) and concentration was measured by a BCA Protein Kit (Cat # 23225, Thermo Fisher). All samples were diluted to the same concentration.

Each protein sample was mixed with 5×SDS protein loading buffer (Cat # BL502A, Biosharp, Hefei, China) and heated at 99°C for 5 min. Protein samples were separated on SDS polyacrylamide gels (Cat # PG112, Epizyme Biotech, Shanghai, China) and transferred to polyvinylidene difluoride (PVDF) membrane (Millipore, Billerica, MA). The PVDF membranes were incubated in 5% BSA (Cat # A8010, Solarbio) or 5% skim milk for 2 h and then incubated in the primary antibody at 4°C for 12 h followed by incubation in horseradish peroxidase (HRP)-conjugated secondary antibodies at room temperature for 1 h. We obtained the following: COX IV (AC610), SQSTM1 (AF5312), ACTB (AF0003), IL-1β (AF7209), IL-18 (AF5207), ASC (AF6234), PINK1 (AF7755), p65(AF1234), Histone H3 (AF0009), BECN1 (AF5123), ATG5 (AF2269), HRP-labeled goat anti-rabbit IgG (H+L) (A0208), HRP-labeled goat anti-mouse IgG (H+L) (A0216) from Beyotime Biotech (Beijing, China). caspase-1 (AF5418), GSDMD (AF4012), NLRP3 (DF7502), parkin (AF0235) from Affinity Biosciences (USA). caspase-11 (ab180673), FOXA2 (ab108422) from Abcam (Cambridge, UK). All antibodies were diluted in Tris-buffered saline/Tween at a ratio of 1:1,000. After extensive washing, the chemiluminescence of bands was detected by a ChemDoc XRS+ Imaging System (Bio-Rad, Hercules, CA, USA) and analyzed with Image J software.

### Enzyme-linked immunosorbent assay

LPS concentrations in rumen fluid and hepatic vein were measured by an LPS ELISA kit (Cat # YH-121619B, Yihe Biotech, Shanghai, China). 10 μL of sample, 40 μL of sample dilution, and 100 μL of HRP-labelled anti-LPS antibody were added to the ELISA plate and incubated at 37°C for 60 min. After washing 5 times, buffer A and buffer B were added to the ELISA plate followed by incubation at 37°C for 15 min. Then, 50 μL of stop buffer was added and the absorbance was measured immediately at 450 nm with a microplate reader (Thermo Fisher).

### Caspase-1 activity analysis

A caspase-1 activity assay kit (Cat # C1101, Beyotime Biotech, Beijing, China) was used for the analysis of hepatic caspase-1 activity. The experiment was conducted according to Manufacturer’s instructions. Briefly, 5 mg of tissue powder and 100 μL of lysate were mixed and fully homogenized on ice. After centrifugation (16 000×g, 4°C, 15 min), 50 μL of supernatant, 40 μL of buffer, and 10 μL of Ac-YVAD-pNA were mixed and incubated at 37°C for 60 min. The absorbance was measured at 405 nm using a microplate reader (Thermo Fisher).

### Hematoxylin-eosin staining

The liver tissue was cut into small pieces and stored in 4% paraformaldehyde (PFA). After being embedded in paraffin, they were cut into 5 μm slices. After dewaxing, they were stained with hematoxylin and eosin.

### ROS activity analysis

The ROS was detected with a dihydroethidium (DHE) kit supplied by Yeasen Biotech (Shanghai, China). Briefly, the frozen liver samples were cut into slices with a cryostat (CM1950, Leica, Germany). Each frozen slice was incubated with 200 μL of 10 μM DHE solution at 37°C for 60 min. After washing with PBS 3 times, the fluorescence was detected and quantitated by LSM 710 confocal laser microscope system (Zeiss, Oberkochen, Germany).

### Statistical analysis

All statistical analyses were performed using SPSS 26.0 (IBM Inc., New York, USA). Results were expressed as the mean and standard error of the mean (mean ± SEM). Rumen fluid pH value was analyzed using Two-way analysis of variance (ANOVA) with a univariate general linear model. All other data were analyzed using One-way ANOVA with the Turkey *post hoc* test. The residuals for each variable were used to assess normality. The data were considered statistically significant at *P*<0.05.

## Results

### Rumen fluid pH and LPS concentrations in rumen fluid and hepatic vein

**Figure 1:**
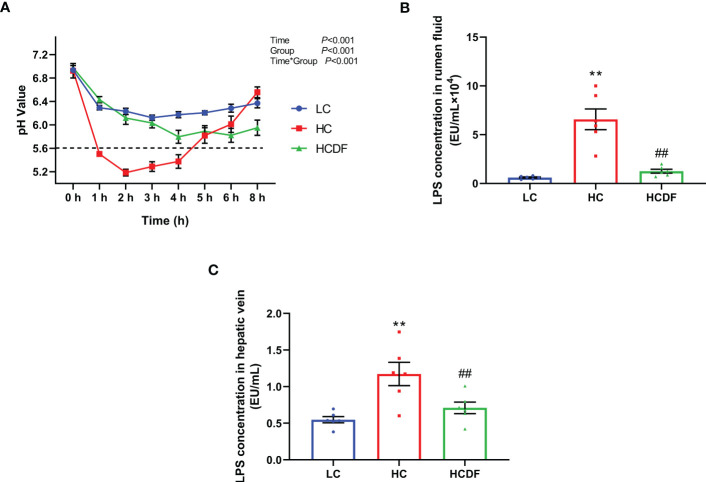
Effect of DF supplementation on ruminal pH and LPS contents in serum and rumen fluid. **(A)** Ruminal pH was detected at different times after feeding. LPS contents in **(B)** rumen fluid and **(C)** hepatic vein were determined. n=6 per group and the values are the mean ± SEM. ^**^ *P*<0.01 vs. LC group; ^##^ *P*<0.01 vs. HC group.

As shown in **Figure 1A**, the rumen fluid pH values were lower in the HC group than the LC group at 1, 2, 3, 4, 5, and 6 h after feeding while the DF supplementation increased the rumen fluid pH value compared with that in HC group. The rumen fluid pH values in the HC group at 1, 2, 3, and 4 h were lower than 5.6, whereas the pH values at all times were above 5.6 in the LC group and HCDF group.

The concentrations of LPS in rumen fluid and hepatic vein were shown in **Figures 1B, C**. Compared with the LC group, LPS concentrations in rumen fluid (*P*<0.001) and hepatic vein (*P*<0.001) were significantly increased in the HC group while DF supplementation significantly decreased high-concentrate diet-induced increased LPS concentrations in rumen fluid (*P*<0.001) and hepatic (*P*<0.001) at different degrees. The data above indicated that feeding a high-concentrate diet induced SARA in the HC group successfully and DF supplementation effectively prevented the occurrence of SARA.

### DF supplementation alleviated SARA-induced liver damage and pyroptosis

**Figure 2:**
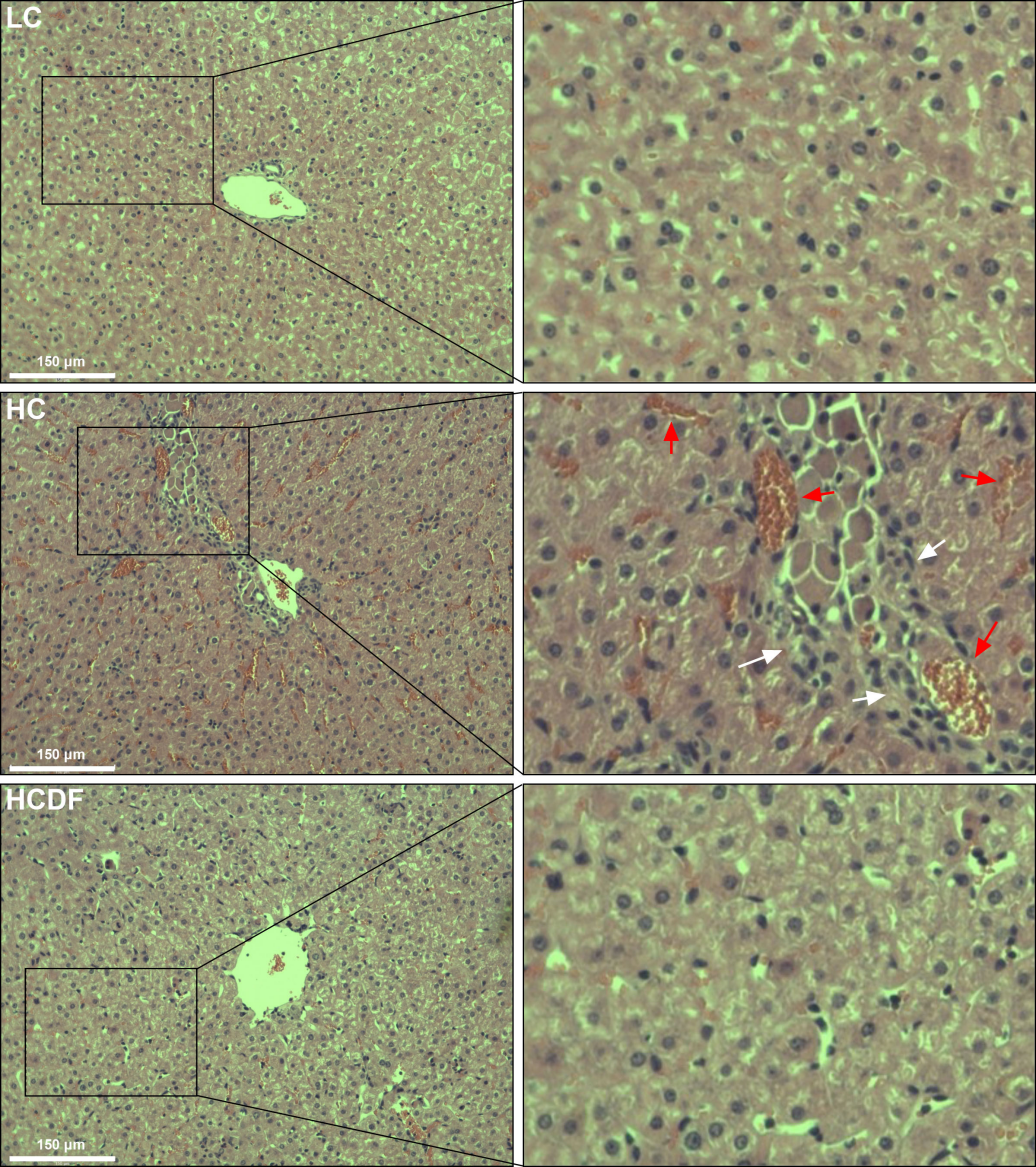
Effect of DF supplementation on SARA-induced liver damage. HE staining was used to detect the histopathology of the liver. Scale bar=150 μm. The white arrows indicate inflammatory cell infiltration, and the red arrows indicate hemorrhage.

**Figure 3:**
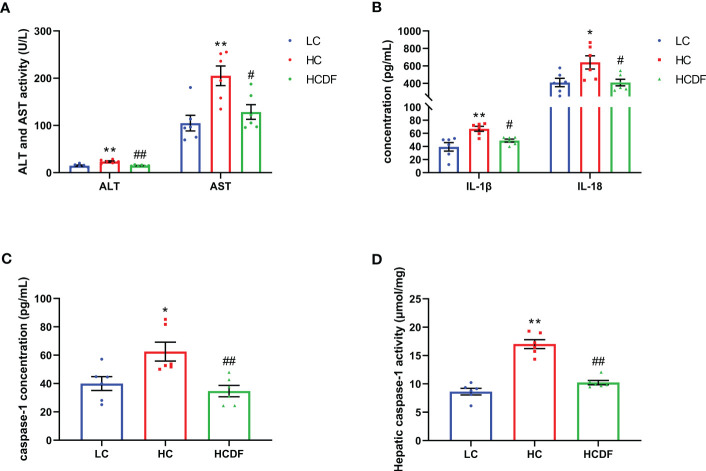
The ALT, AST activity and contents of inflammatory cytokines in hepatic vein. **(A)** The activity of ALT and AST in the hepatic vein. **(B, C)** The contents of IL-1β, IL-18, and caspase-1 in the hepatic vein. **(D)** The caspase-1 activity in liver tissue. n=6 per group and the values are the mean ± SEM. ^*^ *P*<0.05 vs. LC group; ^**^ *P*<0.01 vs. LC group; ^#^ *P*<0.05 vs. HC group; ^##^ *P*<0.01 vs. HC group.

The histopathological evaluation demonstrated that DF supplementation exerted a protective effect against SARA-induced liver damage as evidenced by decreased inflammatory cell infiltration and relieved hemorrhage (**Figure 2**). In addition, the serum alanine aminotransferase (ALT, *P*<0.001) and aspartate aminotransferase (AST, *P*=0.003) activity in the hepatic vein was remarkably elevated in HC group, which was significantly decreased (ALT, *P*=0.001; AST, *P*=0.021) by DF supplementation (**Figure 3A**).

**Figure 4:**
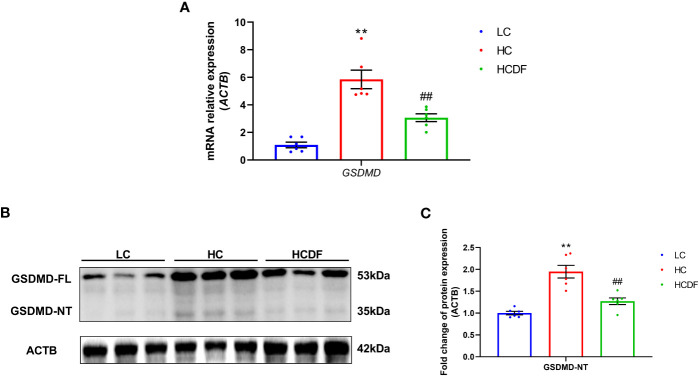
Effect of DF supplementation on SARA-induced pyroptosis in the liver of Hu sheep. The expression of pyroptosis-related **(A)** gene and **(B, C)** protein in the liver tissue of Hu sheep were detected. n=6 per group and the values are the mean ± SEM. ^**^ *P*<0.01 vs. LC group; ^##^ *P*<0.01 vs. HC group.

To evaluate the involvement of pyroptosis in SARA-induced liver damage, we detected pyroptosis-related markers in serum and liver tissue. As shown in **Figures 3B, C**, SARA increased the contents of caspase-1 (*P*=0.022), IL-1β (*P*=0.002), and IL-18 (*P*=0.029) in the hepatic vein while the DF supplementation weakened this increase (caspase-1, *P*=0.005; IL-1β, *P*=0.034; IL-18, *P*=0.029). Consistently, the caspase-1 activity in liver tissue was augmented (*P*<0.001) in the HC group compared with the LC group, while this augmentation was suppressed (*P*<0.001) by DF supplementation (**Figure 3D**). Then, we detected the expression of GSDMD, a critical protein in the process of pyroptosis, in the liver tissue. As shown in **Figure 4**, DF supplementation reversed SARA-induced increased gene expression of *GSDMD* (HC group VS. LC group, *P*<0.001; HCDF group VS. HC group, *P=*0.001) and elevated protein expression of GSDMD-NT (HC group VS. LC group, *P*<0.001; HCDF group VS. HC group, *P*<0.001). The data above demonstrated that DF supplementation attenuated SARA-induced liver damage and pyroptosis in the livers of Hu sheep.

### DF supplementation attenuated SARA-induced activation of NLRP3 inflammasome

**Figure 5:**
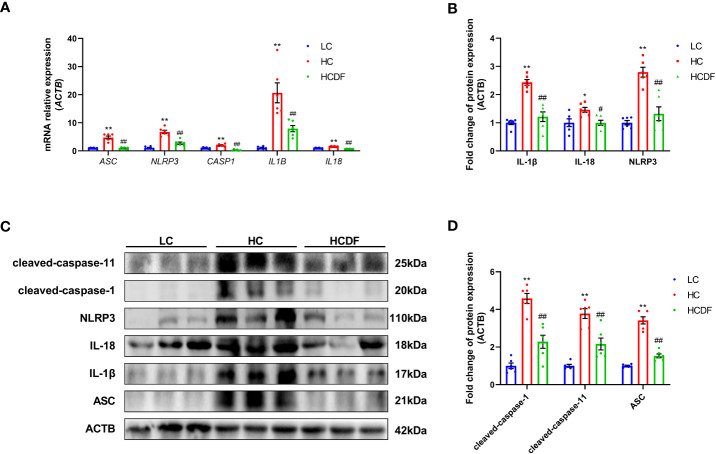
Effect of DF supplementation on the activation of NLRP3 inflammasome. The expression of NLRP3 inflammasome-related **(A)** genes and **(B–D)** proteins in the liver tissue of Hu sheep were detected. n=6 per group and the values are the mean ± SEM. *P<0.05 vs. LC group; ^**^ *P*<0.01 vs. LC group; ^#^ *P*<0.05 vs. HC group; ^##^ *P*<0.01 vs. HC group.

**Figure 6:**
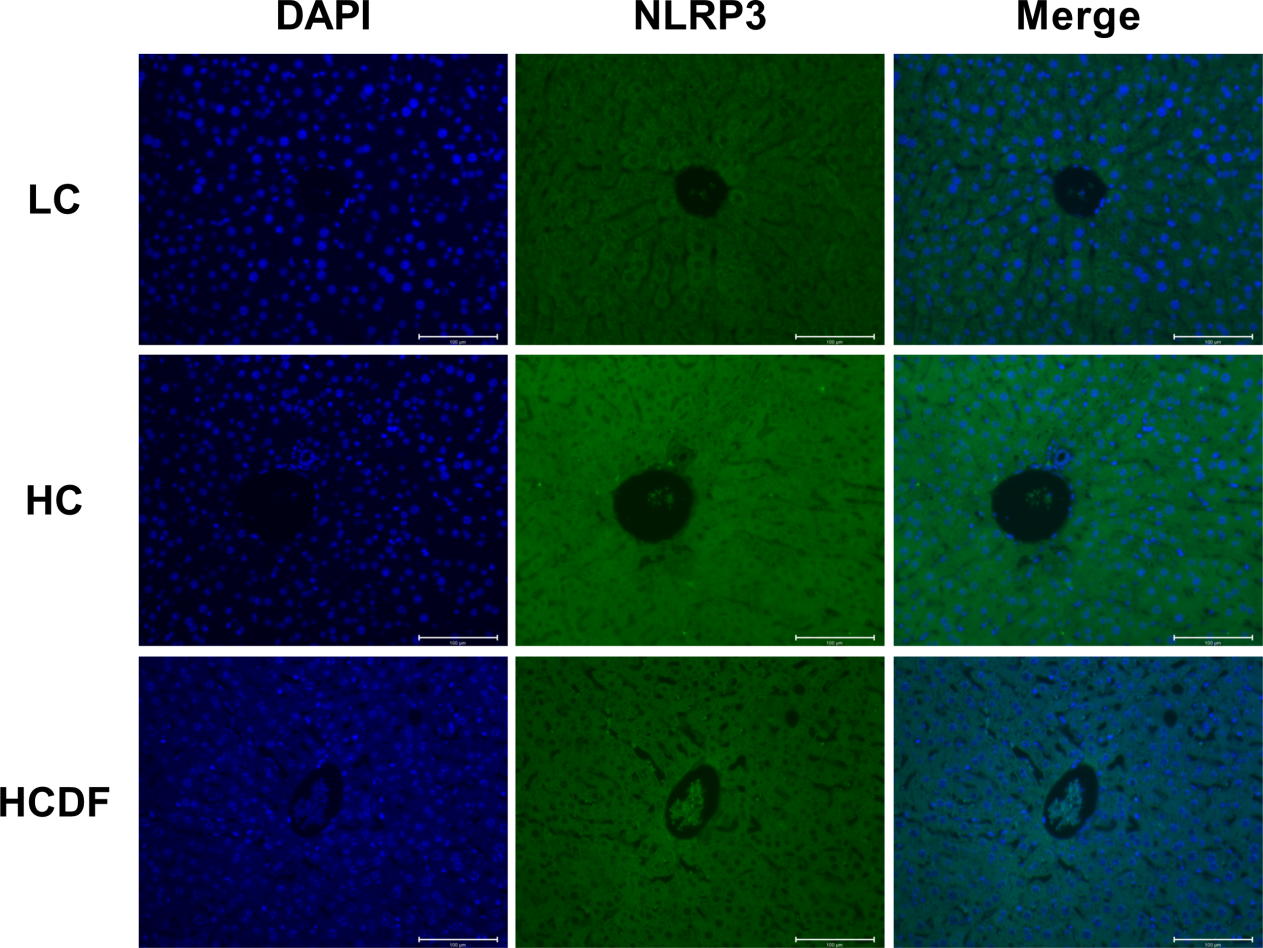
Detection of NLRP3 by immunostaining. The cellular localization of NLRP3 was determined by immunofluorescence staining and visualized by a confocal laser scanning microscope. Scale bar=100 μm.

**Figure 7:**
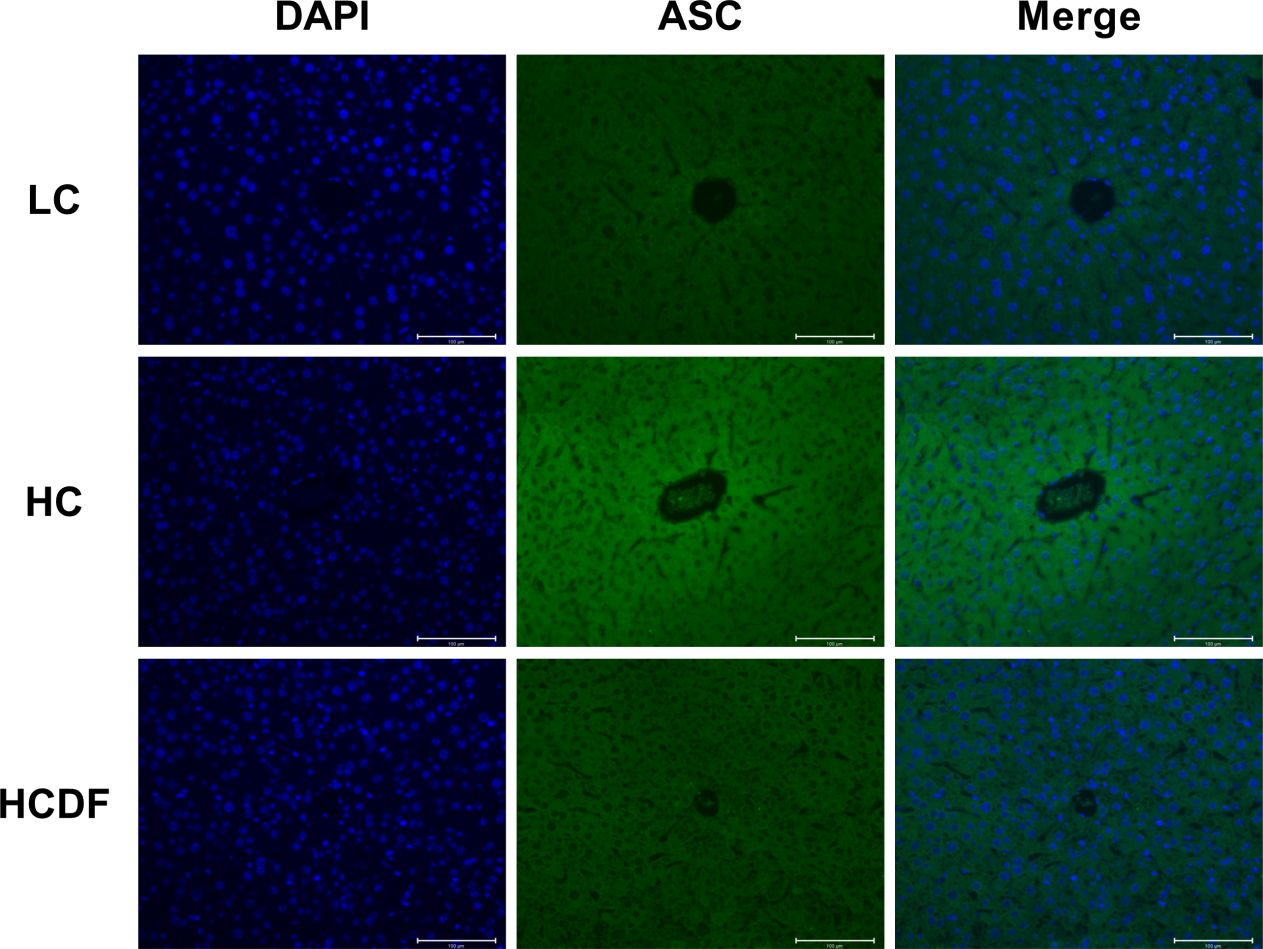
Detection of ASC by immunostaining. The cellular localization of ASC was determined by immunofluorescence staining and visualized by a confocal laser scanning microscope. Scale bar=100 μm.

The pro-inflammatory cytokines- and NLRP3 inflammasome complex-related genes and proteins were detected and results showed that the expression of inflammasome-related genes *ASC* (*P*<0.001), *NLRP3* (*P*<0.001), and *CASP1* (*P*=0.002) and the gene expression of pro-inflammatory cytokines *IL1β* (*P*<0.001) and *IL18* (*P*<0.001) were significantly increased in the HC group compared with the LC group. However, DF supplementation prevented the increased expression of these genes (ASC, *P*<0.001; NLRP3, *P*<0.001; CASP1, *P*<0.001; *IL1β*, *P*=0.002; *IL18*, *P*<0.001) caused by SARA (**Figure 5A**). The result of western blot analysis showed that the protein expression of NLRP3 (*P*<0.001), ASC (*P*<0.001), and cleaved-caspase-1 (*P*<0.001) was dramatically increased in the HC group, while the DF supplementation reversed (NLRP3, *P*<0.001; ASC, *P*<0.001; cleaved-caspase-1, *P*<0.001) this increase (**Figures 5B–D**). Consistently, immunofluorescence analysis showed that SARA increased the contents of NLRP3 and ASC in the livers of Hu sheep, while DF supplementation reversed this increase (**Figures 6** and **7**). In addition, the protein expression of mature IL-1β (*P*<0.001) and mature IL-18 (*P*=0.023), two pro-inflammatory cytokines in the process of pyroptosis, was increased in the HC group. However, DF supplementation decreased the protein expression of mature IL-1β (*P*<0.001) and mature IL-18 (*P*=0.022) compared with those in the HC group (**Figures 5A–C**). Then, we detected the expression of caspase-11, a critical protein involved in the activation of NLRP3 inflammasome in the non-canonical pathway. The results exhibited that the expression of cleaved-caspase-11, the activated form of caspase-11, was increased (*P*<0.001) in the HC group compared with the LC group, while the DF supplementation reversed (*P*=0.01) this increase (**Figures 5A, C**).

### DF supplementation reversed SARA-induced elevated mitophagy in the livers of Hu sheep

**Figure 8:**
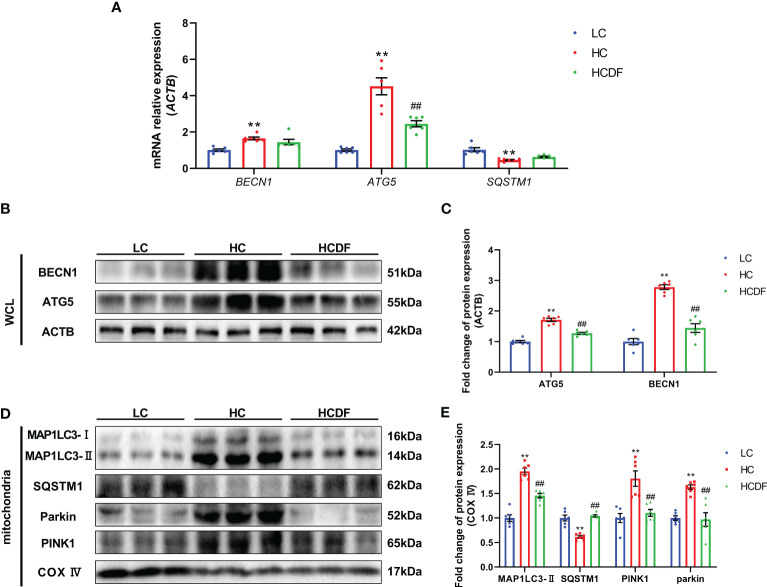
Effect of DF supplementation on mitophagy. **(A)** Expression of autophagy-related genes. **(B, C)** Expression of autophagy-related proteins in the whole cell lysate. **(D, E)** Expression of mitophagy-related proteins in the mitochondria protein extract. n=6 per group and the values are the mean ± SEM. ^**^ *P*<0.01 vs. LC group; ^##^ *P*<0.01 vs. HC group.

**Figure 9:**
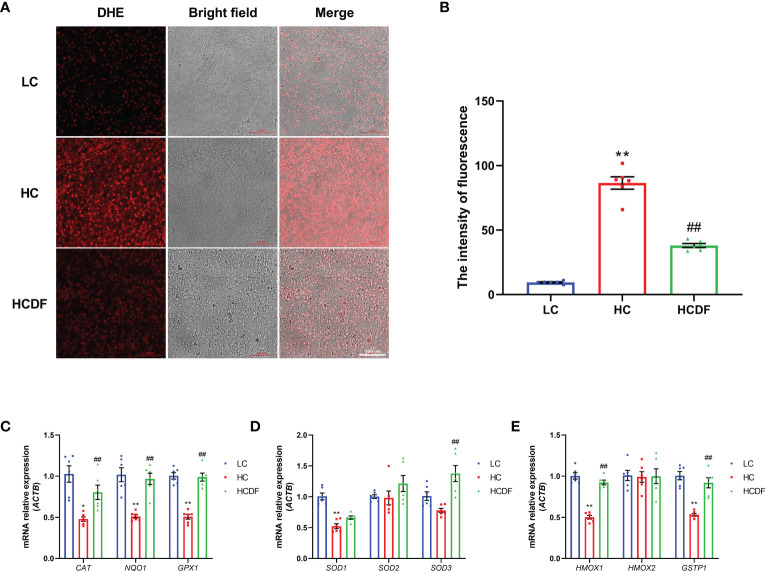
Effect of DF supplementation on ROS production and the expression of antioxidant-related genes. **(A, B)** The effect of DF supplementation on the ROS production was determined by DHE staining and visualized by a confocal laser scanning microscope. **(C-E)** The expression of antioxidant-related genes was determined by qPCR analysis. Scale bar=100 μm. ^**^ *P*<0.01 vs. LC group; ^##^ *P*<0.01 vs. HC group.

To investigate the mitophagy in the livers of Hu sheep, qPCR and western blot analysis were applied. The results were shown in **Figure 8**, SARA increased the expression of autophagy-related genes *BECN1* (*P*<0.001) and *ATG5* (*P*<0.001), and decreased the gene expression of *SQSTM1* (*P*<0.001). However, compared with the HC group, DF supplementation decreased the gene expression of *BECN1* (*P*=0.365) and *ATG5* (*P*<0.001) and increased the gene expression of *SQSTM1* (*P*=0.203) (**Figure 8A**). Consistently, western blot analysis showed that SARA increased the protein expression of BECN1 (*P*<0.001), ATG5 (*P*<0.001) while the DF supplementation reversed (BECN1, *P*<0.001; ATG5, *P*<0.001) this increase (**Figures 8B, C**). To better investigate mitophagy, we extracted mitochondrial protein for western blot analysis. As shown in **Figures 8D, E**, the expression of MAP1LC3-II (*P*<0.001) was increased while the expression of SQSTM1 (*P*<0.001) was decreased in the mitochondria protein extract. However, DF supplementation reversed (MAP1LC3-II, *P*<0.001; SQSTM1, *P*<0.001) these changes. Then, we investigated the expression of Parkin and PIINK1, two proteins responsible for the recognition of substrates in mitophagy. The results showed that the protein expression of Parkin (*P*<0.001) and PINK1 (*P*<0.001) was increased in the HC group compared with the LC group, while DF supplementation decreased the protein expression of Parkin (*P*<0.001) and PINK1 (*P*=0.001) compared with those in the HC group (**Figures 8D, E**). Moreover, the ROS and the expression of antioxidant-related genes were detected by the DHE staining and qPCR analysis. The data showed that ROS activity was significantly increased (*P*<0.001) in the HC group compared with the LC group, while DF supplementation reversed (*P*<0.001) this increase (**Figures 9A, B**). Compared with the LC group, the gene expression of catalase (*CAT)* (*P*=0.001), NAD(P)H quinone dehydrogenase 1 (*NQO1)* (*P*<0.001), glutathione peroxidase 1 (*GPX1)* (*P*<0.001), superoxide dismutase 1 (*SOD1)* (*P*<0.001), heme oxygenase 1 (*HMOX1)* (*P*<0.001), and glutathione S-transferase pi 1 (*GSTP1)* (*P*<0.001) was significantly decreased in the HC group, while DF supplementation inhibited this decrease (**Figures 9C-E**). Taken together, these results demonstrated that SARA enhanced mitophagy in the livers of Hu sheep, which was alleviated by DF supplementation.

### DF supplementation inhibited SARA-induced the activation of TLR4-NF-κB signaling pathway

**Figure 10:**
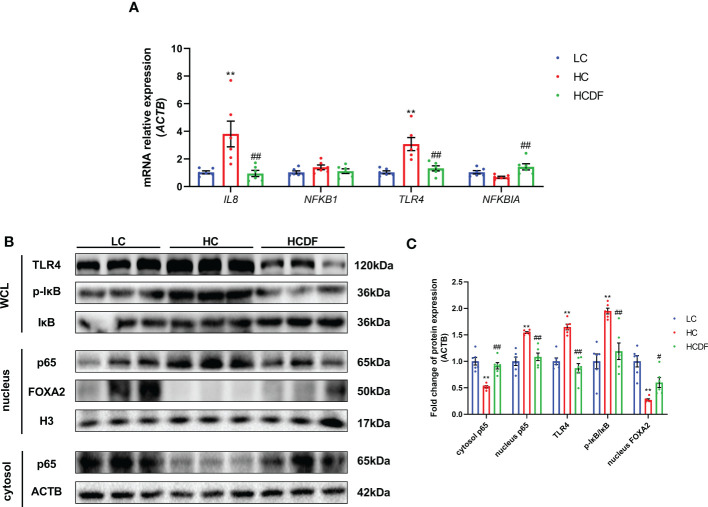
Effect of DF supplementation on the activation of the NF-κB signaling pathway. The expression of TLR4-NF-κB pathway-related **(A)** genes and **(B, C)** proteins in the liver tissue of Hu sheep was detected. n=6 per group and the values are the mean ± SEM. ^**^ *P*<0.01 vs. LC group; ^#^ *P*<0.05 vs. HC group; ^##^ *P*<0.01 vs. HC group.

**Figure 11:**
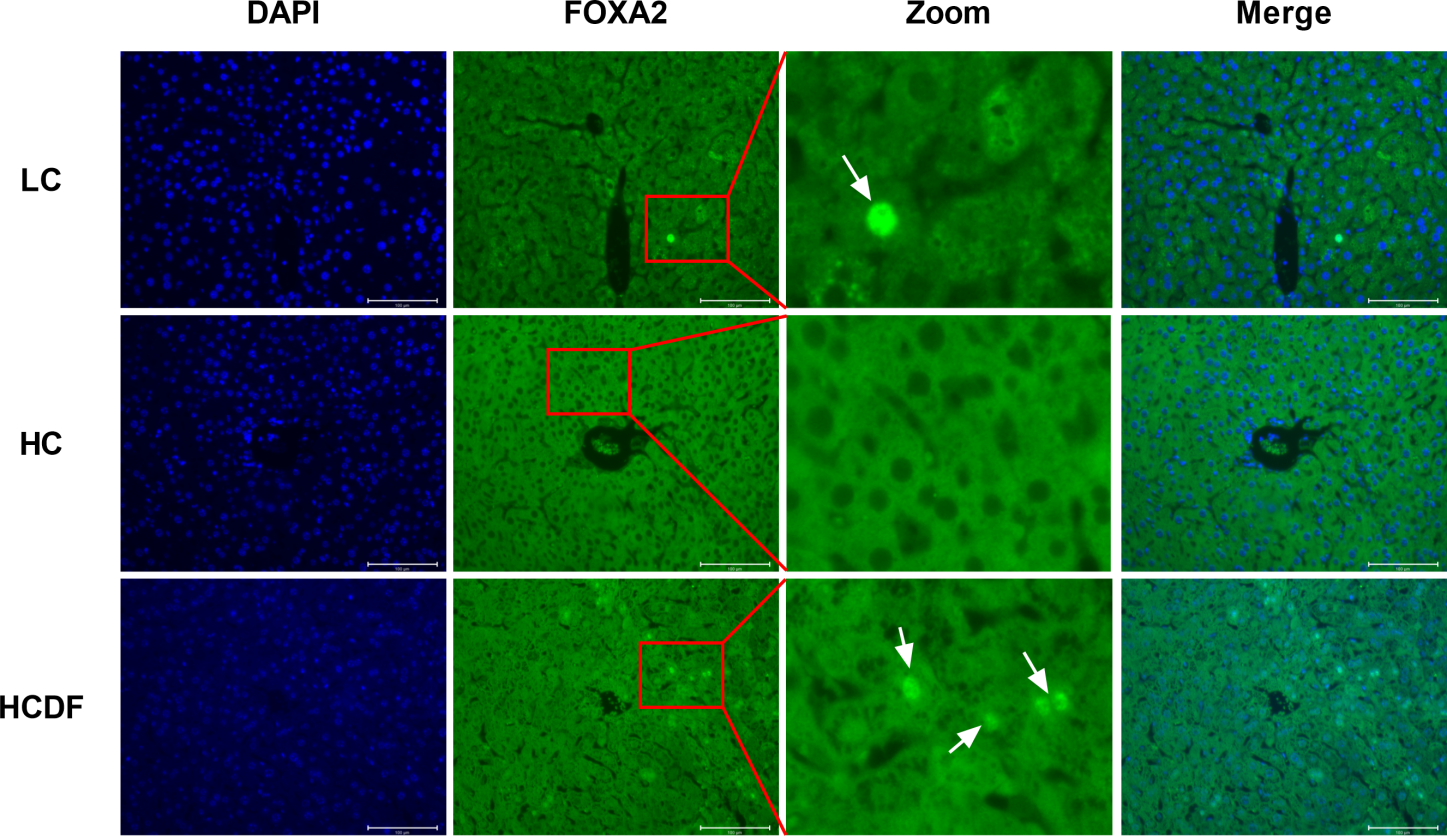
Detection of cellular localization of FOXA2 by immune staining. The cellular localization of FOXA2 was determined by immunofluorescence staining and visualized by a confocal laser scanning microscope. Scale bar=100 μm. The arrows indicate FOXA2 in the nucleus.

As shown in **Figure 10**, the gene expression of *IL8* (*P*=0.008) and *TLR4* (*P*=0.001) was significantly increased in the HC group compared with the LC group. However, the DF supplementation decreased gene expression of *IL8* (*P*=0.006) and *TLR4* (*P=*0.002) compared with those in the HC group (**Figure 10A**). The western blot analysis showed that compared with the LC group, the protein expression of TLR4 (*P*<0.001) and nucleus p65 (*P*<0.001) and the ratio of p-IκB/IκB (*P*<0.001) were significantly increased in the HC group, which was reversed (TLR4, *P*<0.001; p65, *P*<0.001; p-IκB/IκB, *P*=0.002) by DF supplementation (**Figures 10B, C**). Moreover, we detected forkhead box A2 (FOXA2), a critical transcription factor enriched in the liver. The western blot (**Figures 10B, C**) and immunofluorescence (**Figure 11**) analysis showed that SARA induced decreased FOXA2 in the nucleus, while DF supplementation restored the content of FOXA2 in the nucleus. These results demonstrated that DF supplementation suppressed the activation of the TLR4-NF-κB signaling pathway and promoted the entry of FOXA2 into the nucleus.

## Discussion

In this study, feeding a high-concentrate diet decreased rumen fluid pH, which meets the criteria for SARA occurrence, i.e. rumen pH value below 5.6 for more than 3 h per day (1, 22). DF supplementation increased rumen fluid pH value, decreased ruminal LPS, and enabled sheep to effectively avoid SARA. Meanwhile, SARA induced inflammatory cell infiltration and hemorrhage in liver tissue as well as increased AST and ALT activity in the hepatic vein, which was alleviated by DF supplementation.

Disordered ruminal microbial community occurs in animals with long-term SARA. Low rumen fluid pH causes lysis of acid-labile bacteria in the rumen, leading to the release of a large amount of LPS and disruption of rumen epithelium integrity (23, 24). Liver is the “first pass” organ and is continually invaded by microbial particles from the digestive system (25). Rumen-derived LPS could reach the liver through the portal vein, causing liver damage (26, 27). In our study, DF was selected as a feed additive for its properties that low in cost, has weak buffering capacity and could regulate ruminal microbiota. As a strong alkali and weak acid salt, DF itself has a weak buffering capacity and could neutralize the organic acid in the rumen. Moreover, DF could increase rumen pH by regulating ruminal microbiota. It has been reported that DF could promote the growth of lactic acid-utilizing bacteria such as *Selenomonas ruminantium* and inhibit the growth of lactic acid-synthesizing bacteria such as *Streptococcus bovis* (20, 28). Yang et al. reported that DF supplementation in high-concentrate diet significantly increased rumen pH and the abundance of *Selenomonas ruminantium* (29). Jin et al. also reported a remarkably increased rumen pH in an *in vitro* fermentation system (19). Therefore, we supposed that DF modified the ruminal microbial community and promoted the growth of lactic acid-utilizing bacteria, which prevented the decrease of rumen fluid pH and attenuated LPS release. In our experiment, we choose a dose of 10 g/d DF according to a previous study (29). The results confirm our point. The DF supplementation significantly increased rumen fluid pH value. The rumen pH values at all time points were above 5.6 in the HCDF group. Moreover, the decreased LPS content in the hepatic vein was detected in the HCDF group, identifying LPS as a potential factor in causing liver damage. However, the alteration of rumen microbial composition and the abundance of lactic acid-utilizing bacteria among the three groups were not investigated, which is a limitation of this study.

In our previous studies, we found that SARA could induce oxidative stress, inflammatory responses, and apoptosis in hepatocytes, resulting in liver damage (21, 26, 30). However, pyroptosis, a novel programmed cell death, has not been investigated in ruminants with SARA. Accumulating evidence indicated that pyroptosis is an important factor leading to tissue damage. Qu et al. reported that deoxynivalenol induced intestinal epithelial cells pyroptosis and enteritis in mice, and inhibiting pyroptosis promoted the viability of enterocyte (31). Han et al. revealed that pyroptosis was involved in LPS-induced neuronal injury (16). When pyroptosis occurs, the cell membrane is ruptured and pro-inflammatory cytokines such as IL-1β and IL-18 are released to extracellular space, leading to tissue inflammatory damage (32). Cell membrane rupture depends on the formation of the GSDMD-NT oligomer. The full-length GSDMD protein consists of GSDMD-NT and GSDMD-CT, and GSDMD-CT has an inhibitory effect on GSDMD-NT. When the full-length GSDMD is cleaved into GSDMD-NT and GSDMD-CT, GSDMD-NT is activated. GSDMD-NT oligomerizes and binds to the cell membrane, which depends on its lipid-binding capacity (8). GSDMD-NT oligomer could bind to the inner cell membrane and causes the formation of pores with a diameter less than 10-13 nm to let IL-1β, IL-18, and inflammasome complex particles pass through (16). In our study, we found that the protein expression of GSDMD-NT in the liver and contents of IL-1β and IL-18 in the hepatic vein were elevated in the HC group, which indicated aggravated hepatocyte pyroptosis. DF supplementation inhibited this aggravation. Therefore, we confirm that DF supplementation inhibited SARA-induced pyroptosis in the livers of Hu sheep.

The NLRP3 inflammasome is vital for the onset of pyroptosis. It has been demonstrated that using MCC950, a specific antagonist of NLRP3 inflammasome, inhibited pyroptosis (31, 33). There are two ways to activate NLRP3 inflammasome, i.e. canonical and non-canonical pathways. In the canonical pathway, PAMPs and DAMPs make NLPR3, ASC, and pro-caspase-1 form the NLRP3 inflammasome complex (34), leading to the activation of pro-caspase-1. The activated caspase-1 could cleave pro-IL-1β, pro-IL-18, and full-length GSDMD into mature IL-1β, mature IL-18, GSDMD-CT, and GSDMD-NT, leading to pyroptosis (35). In the non-canonical pathway, gram-negative bacteria and LPS could activate caspase-11 (9), and the activated caspase-11 cleaves the full-length GSDMD into GSDMD-NT and GSDMD-CT. In addition to being the element of the GSDMD-NT oligomer, GSDMD-NT leads to the formation of NLRP3-ASC-pro-caspase-1 inflammasome complex, inducing the cleavage of pro-IL-1β and pro-IL-18 into mature IL-1β and mature IL-18 and triggering pyroptosis. In our study, we confirmed that DF supplementation inhibited SARA-induced activation of NLRP3 inflammasome as evidenced by decreased gene and protein expression of NLRP3, ASC, and caspase-1. In addition, we found that feeding a high-concentrate diet increased cleaved-caspase-11 in protein expression, which indicated the involvement of the non-canonical pathway. The accumulating LPS caused by the lysis of gram-negative bacteria in the rumen reached the liver through damaged rumen epithelium and activated NLRP3 inflammasome *via* non-canonical pathway, thus leading to hepatocyte pyroptosis.

Numerous studies reported that disturbed mitophagy could induce ROS accumulation, which is crucial for the activation of NLRP3 inflammasome. It has been demonstrated that mitochondria damage caused by LPS led to excessive ROS, causing the activation of NLRP3 inflammasome (13). Moreover, Liu et al. found inhibiting mitophagy by using mitophagy antagonist CsA increased ROS and led to NLRP3 inflammasome activation (14). These studies raised our interest in whether NLRP3 inflammasome activation is related to disturbed mitophagy and increased ROS in ruminants with SARA. Therefore, we further investigate the mitophagy and the activity of ROS in the liver. Mitochondria protein was extracted to detect the expression of mitophagy-related proteins MAP1LC3-II, SQSTM1, PINK1, and Parkin. PINK1-Parkin-mediated mitophagy has been widely investigated. When mitochondria are damaged, PINK1 is recruited to the outer membrane of mitochondria and forms diners, leading to the activation of Parkin (36, 37). The activated Parkin can ubiquitylate a large number of proteins on the surface of mitochondria, which could be recognized by autophagy adaptors such as SQSTM1, NBR1, NDP52, TAX1BP1, and OPTN, inducing phagocytosis of damaged mitochondria by autophagosome (38). The increased ROS and decreased gene expression of *CAT*, *NQO1*, *GPX1*, *SOD1*, *HMOX1*, and *GSTP1* in the liver were detected in the HC group, indicating ROS as a vital factor for NLRP3 inflammasome activation and the occurrence of oxidative stress in the liver. Meanwhile, We found enhanced mitophagy in the HC group as evidenced by increased protein expression of MAP1LC3-II, PINK1, and Parkin and decreased protein expression of SQSTM1. This may be due to the adaptive enhancement of mitophagy to maintain cellular homeostasis in face of increased intracellular ROS. However, the mitophagy and ROS activity were attenuated in the HCDF group. DF supplementation decreased the amount of LPS entering the liver and made hepatocytes suffer a less serious stimulation. Thus, mitophagy did not need to be maintained at a high level.

Transcription factor NF-κB is not directly involved in the activation of NLRP3 but acts as a second signal to regulate the transcription of inflammasome- and pyroptosis-related genes such as *GSDMD*, *CASP1*, *NLRP3*, *IL1B*, and *IL18* (39). In our previous study, we found FOXA2, a liver-enriched transcription factor, could interact with NF-κB. NF-κB could bind to the promoter region of FOXA2, leading to the inhibited activity of FOXA2 (40). In addition, overexpression of FOXA2 could suppress LPS-induced activation of NF-κB in bovine primary hepatocytes (41). In this study, we detected activated NF-κB signaling pathway and decreased nucleus FOXA2 in the HC group, suggesting that inhibition of FOXA2 may facilitate up-regulated NF-κB signaling pathway and SARA-induced hepatocyte pyroptosis. However, DF supplementation inhibited the activation of the NF-κB signaling pathway and increased the protein expression of nucleus FOXA2.

## Conclusions

Feeding a high-concentrate diet induced SARA and increased the concentration of LPS reaching the liver, disrupted mitophagy homeostasis, and subsequently led to increased intracellular ROS and activation of NLRP3 inflammasome, ultimately leading to hepatocytes pyroptosis and inflammatory damage to the liver. DF supplementation could increase rumen fluid pH value by regulating ruminal microbiota, thus inhibiting the lysis of gram-negative bacteria and decreasing LPS concentration, which reduced pathogen invasion to the liver and protected the liver from damage.

## Data availability statement

The original contributions presented in the study are included in the article/**Supplementary Material**. Further inquiries can be directed to the corresponding author.

## Ethics statement

The animal study was reviewed and approved by Animal Care and Use Committee of Nanjing Agricultural University (NJAU.No20220112003).

## Author contributions

HZ performed the whole experiments and wrote the manuscript. HZ, SZ, GW, and WX performed the animal experiment. HS participated in performing the western blot analysis of this study. MM, GC, and XS performed manuscript review. XS provided the funding. All authors contributed to the article and approved the submitted version.
