# Supplementary material for: Dietary disodium fumarate supplementation alleviates subacute ruminal acidosis (SARA)-induced liver damage by inhibiting pyroptosis via mitophagy-NLRP3 inflammasome pathway in lactating Hu sheep
Source: Front Immunol. 2023 May 19;14:1197133. doi: 10.3389/fimmu.2023.1197133 (PMC10235698; doi:10.3389/fimmu.2023.1197133)

GSDMD-FL and GSDMD-NT


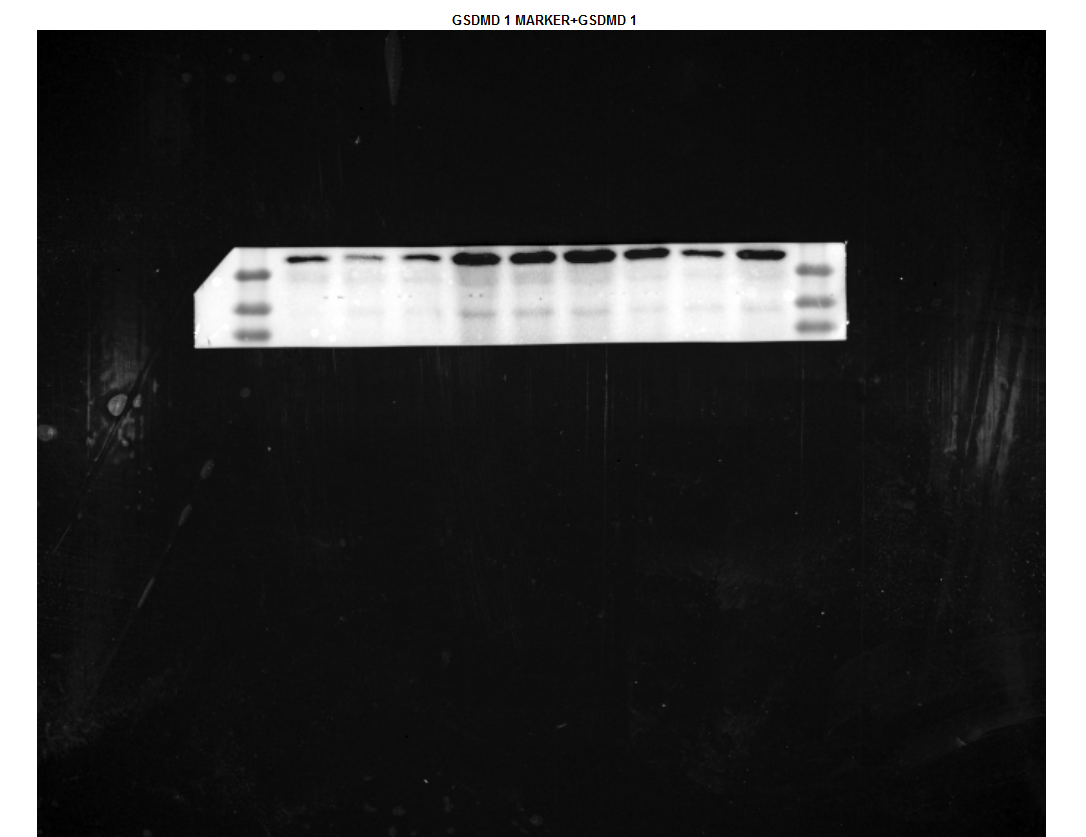


ACTB


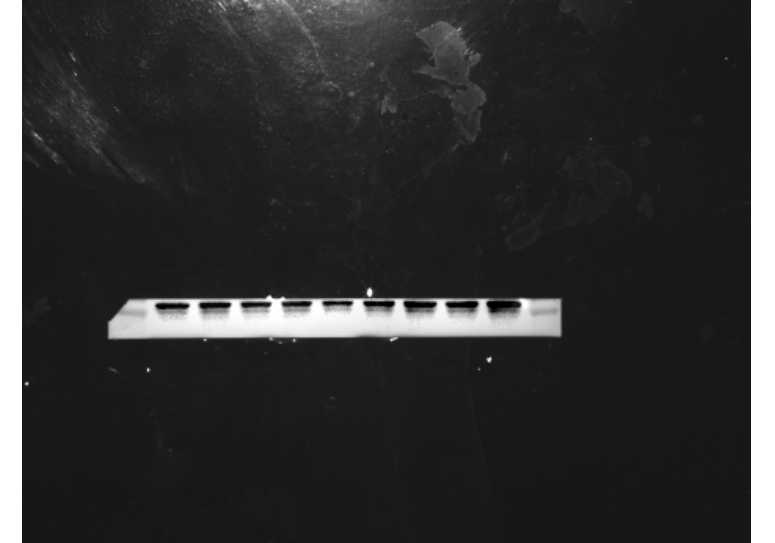


Cleaved-caspase-11


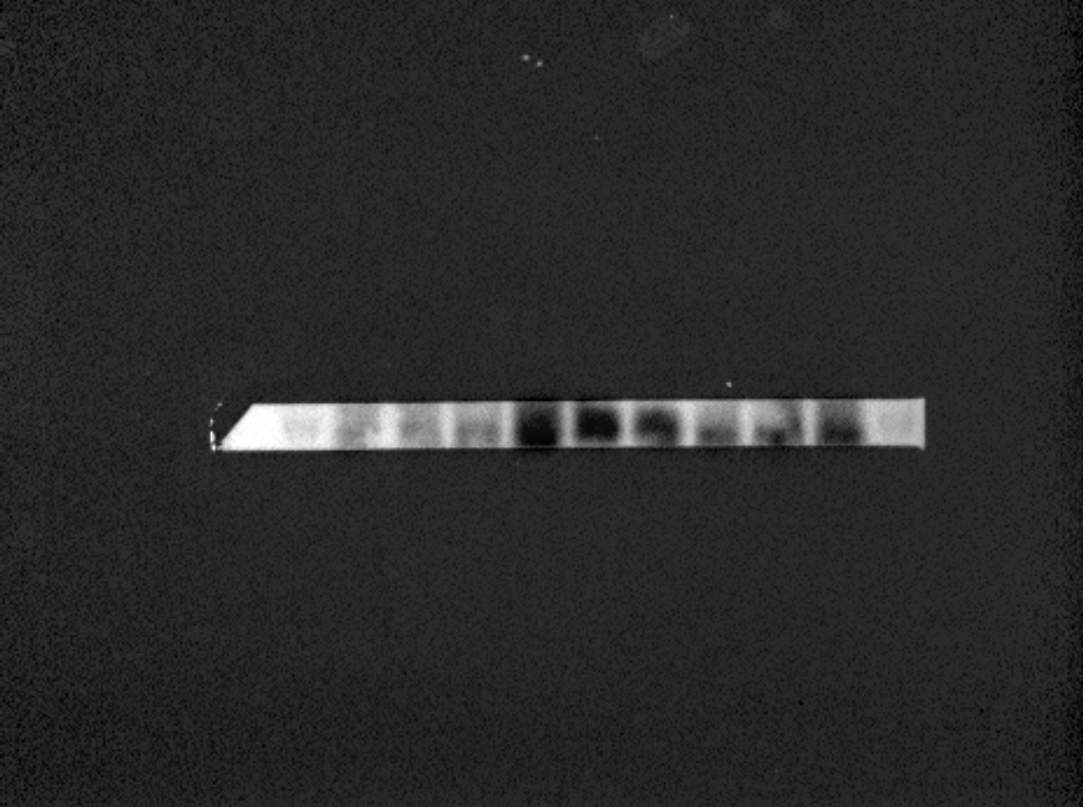


Cleaved-caspase-1


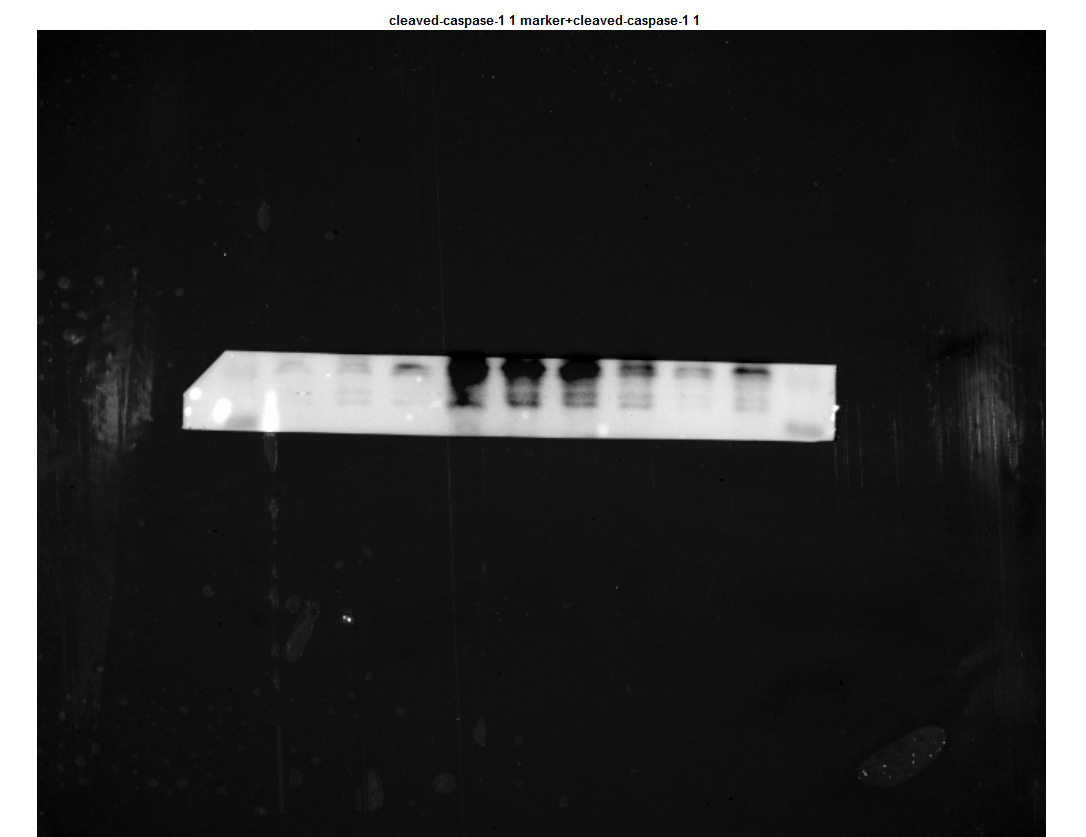


NLRP3


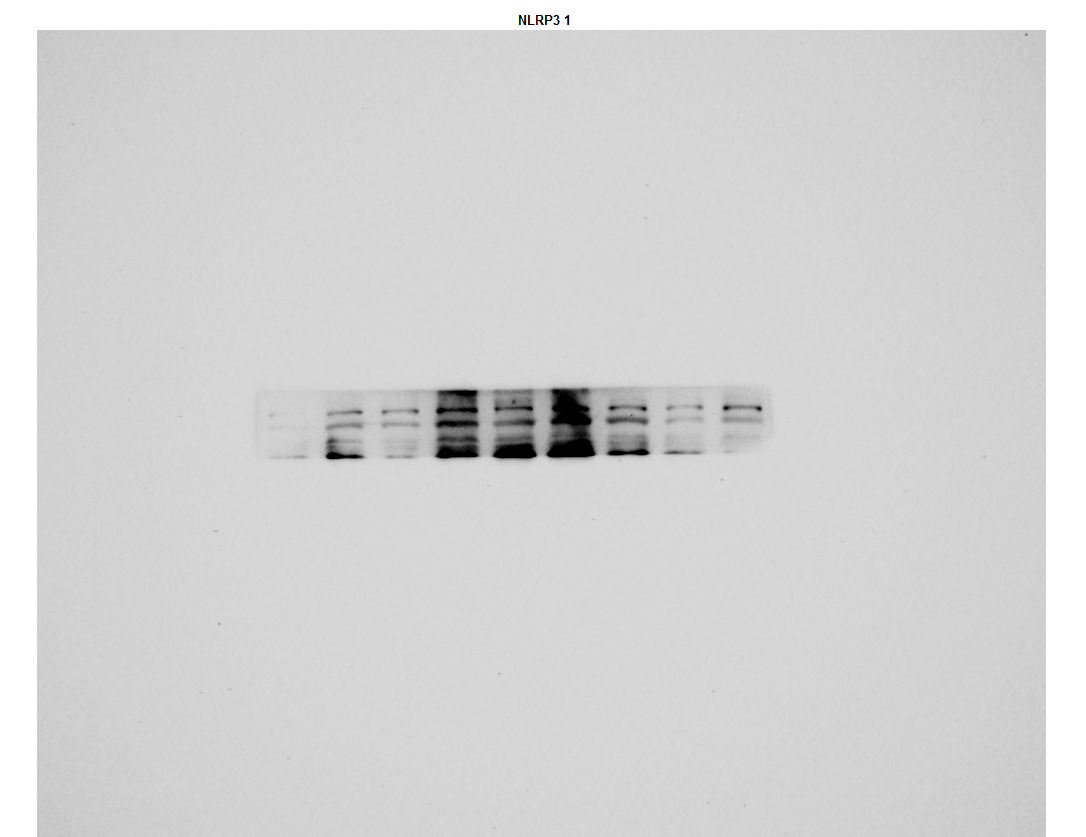


IL-18


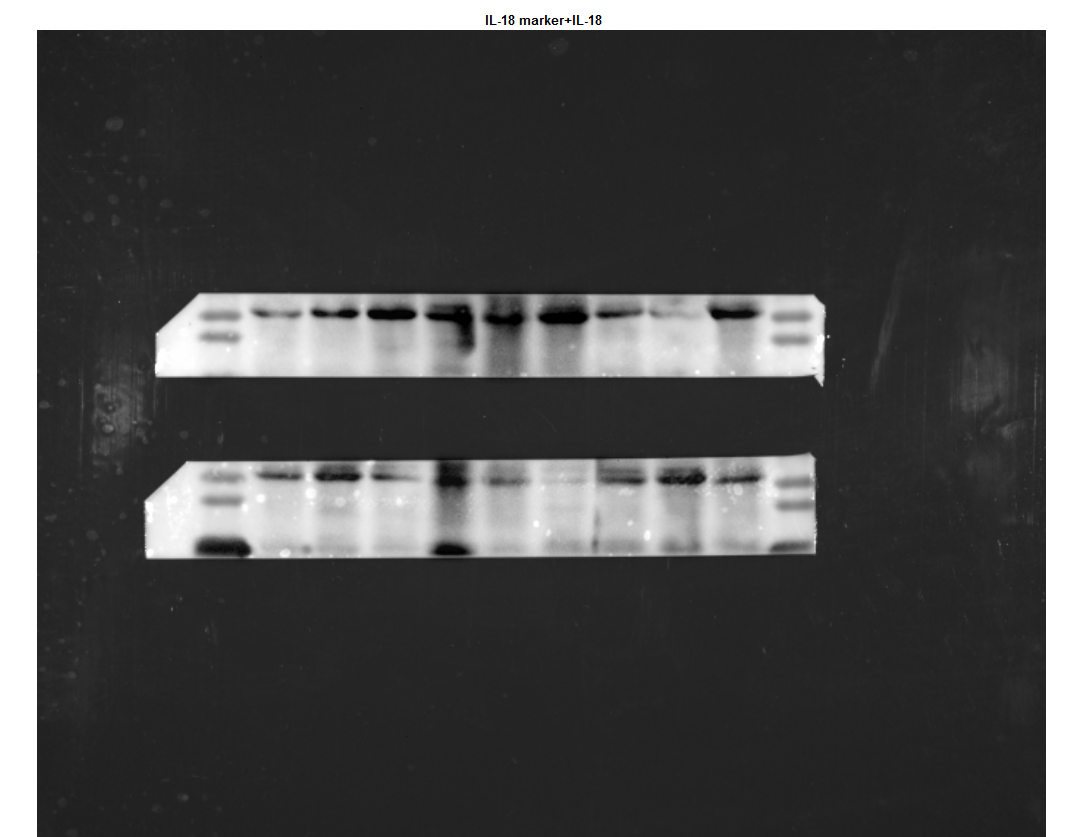


IL-1β


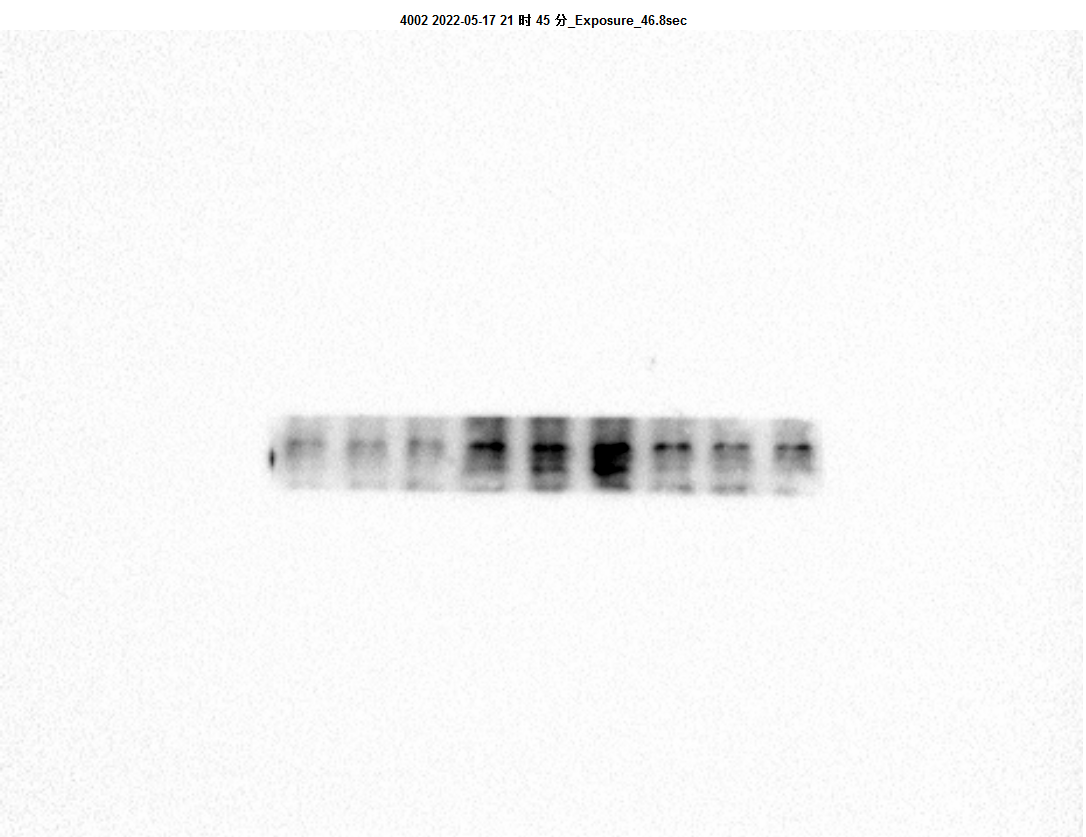


ASC


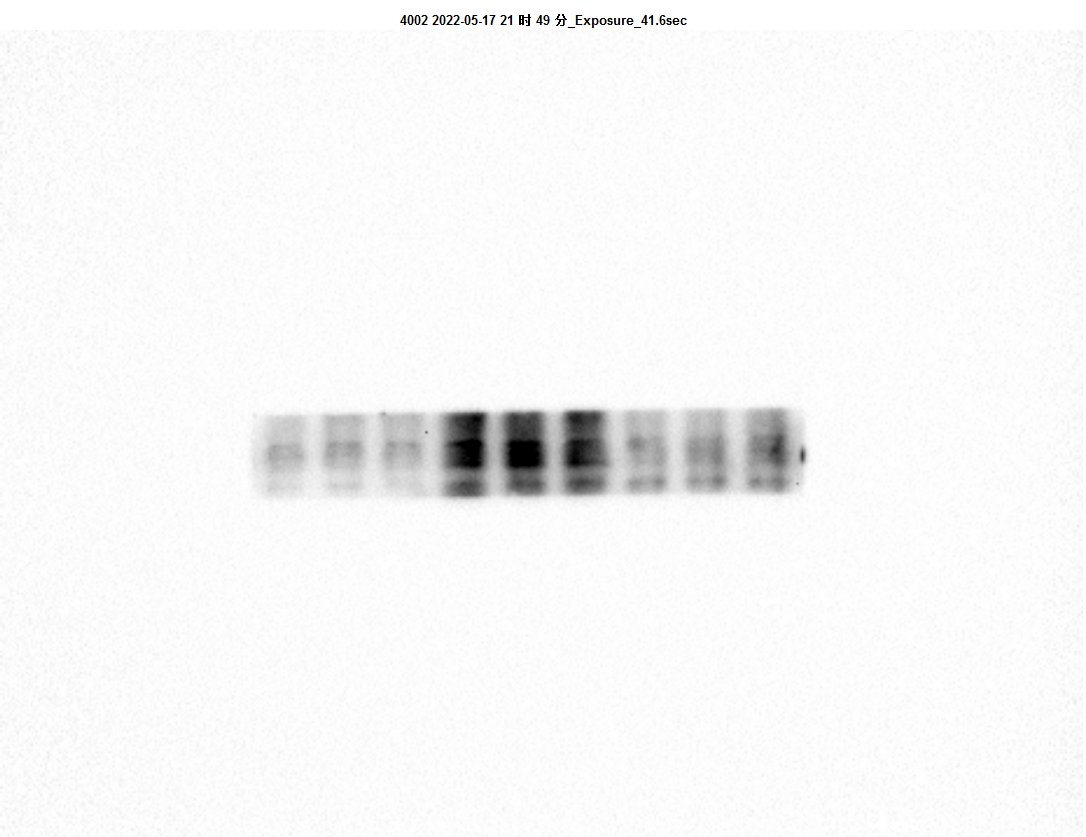


ACTB


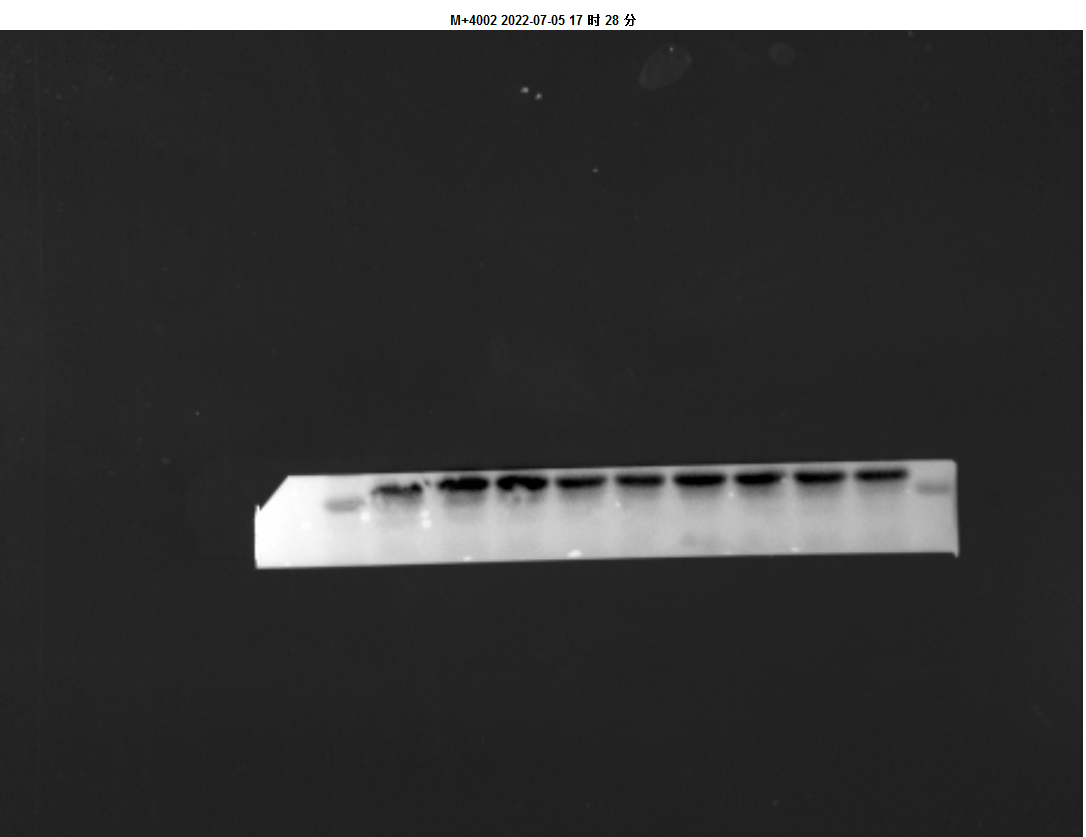


BECN1


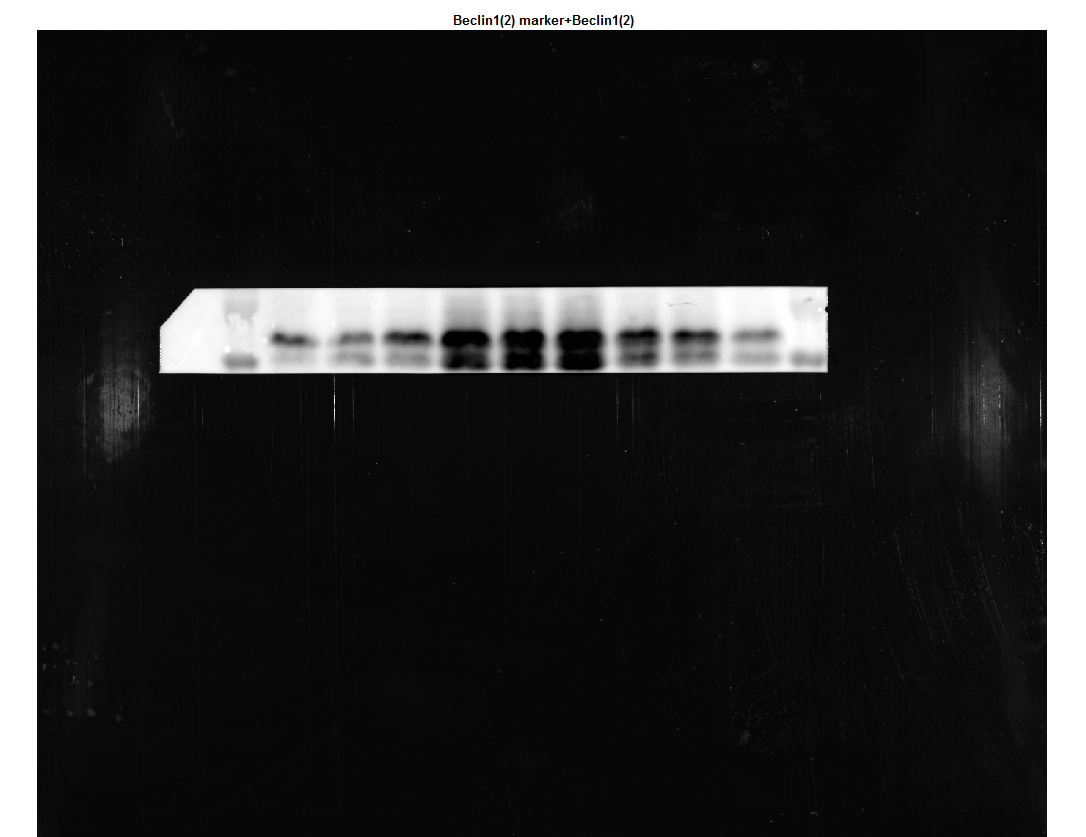


ATG5


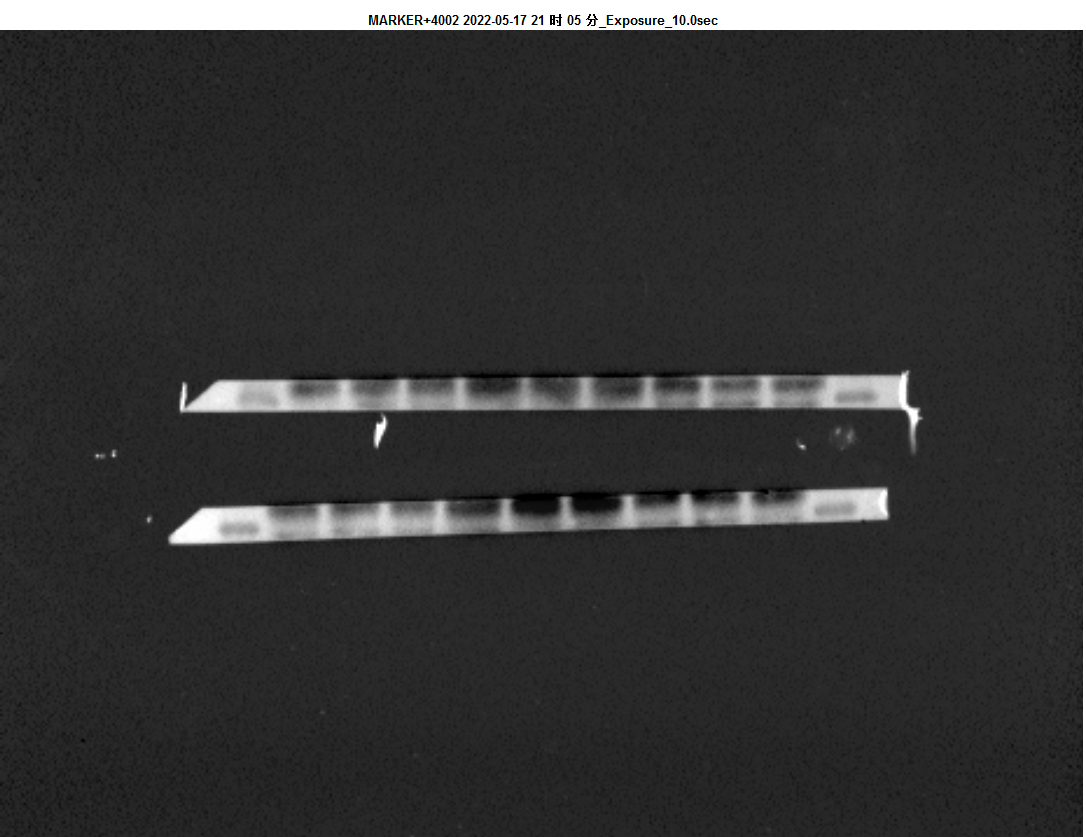


ACTB


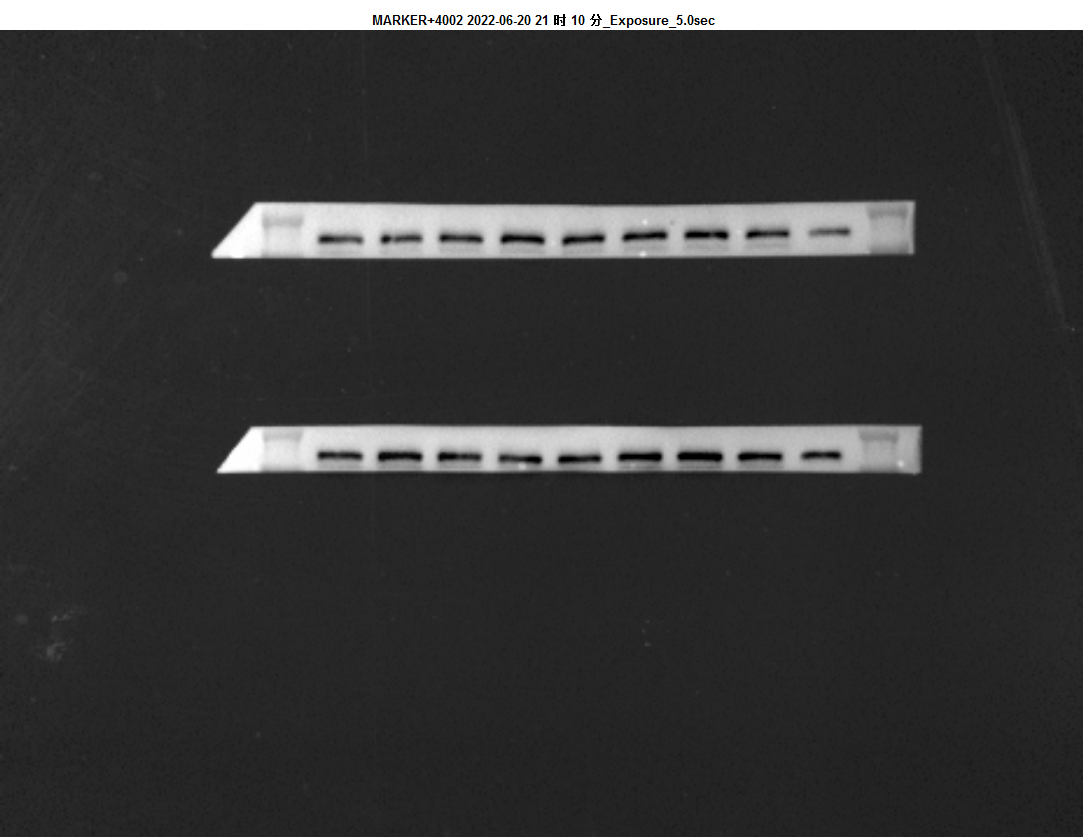


MAP1LC3


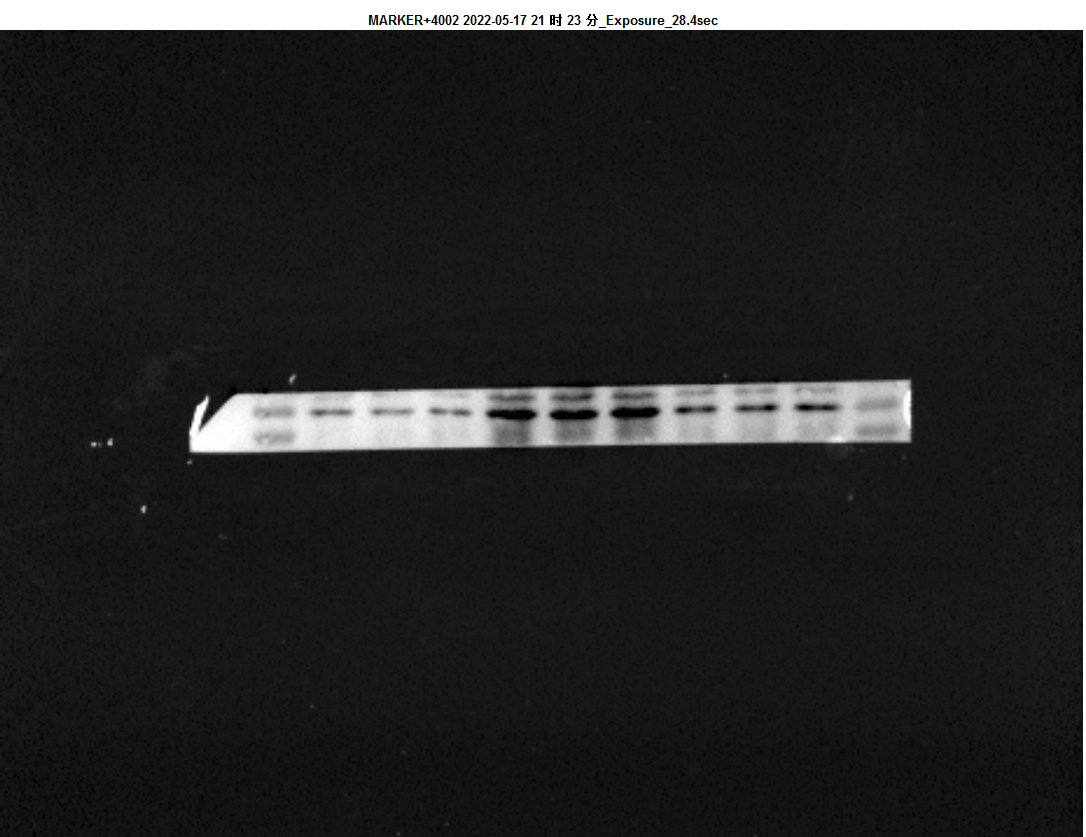


SQSTM1


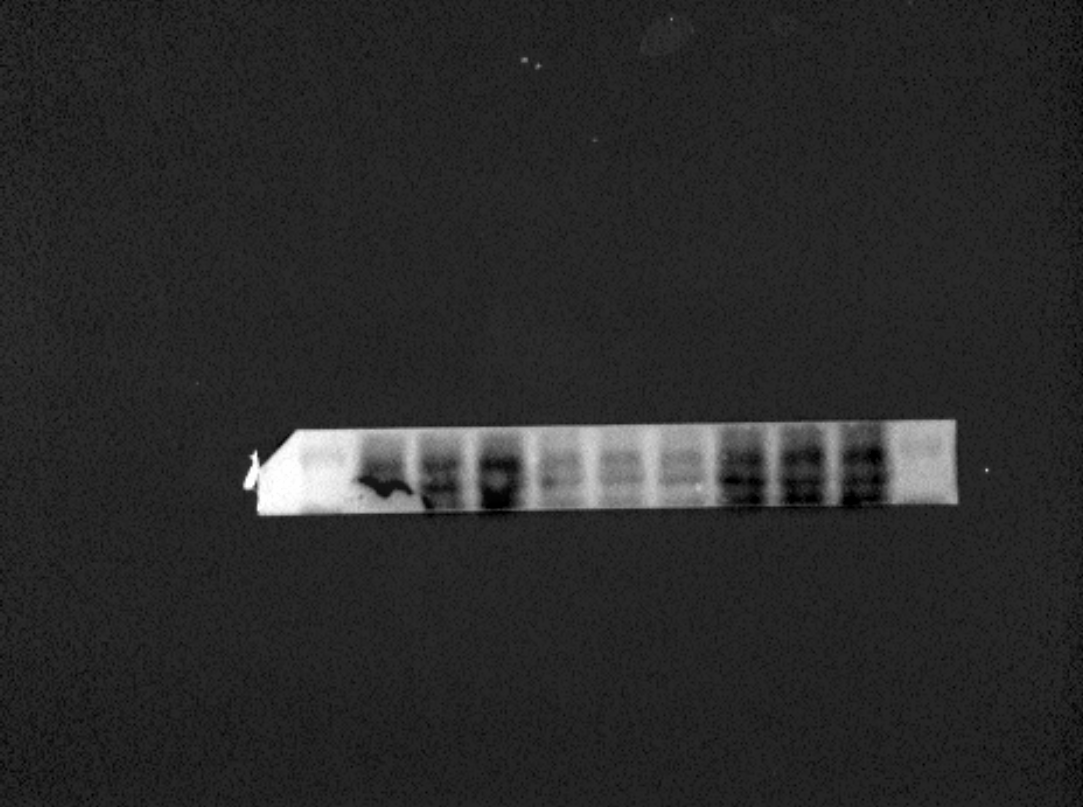


Parkin


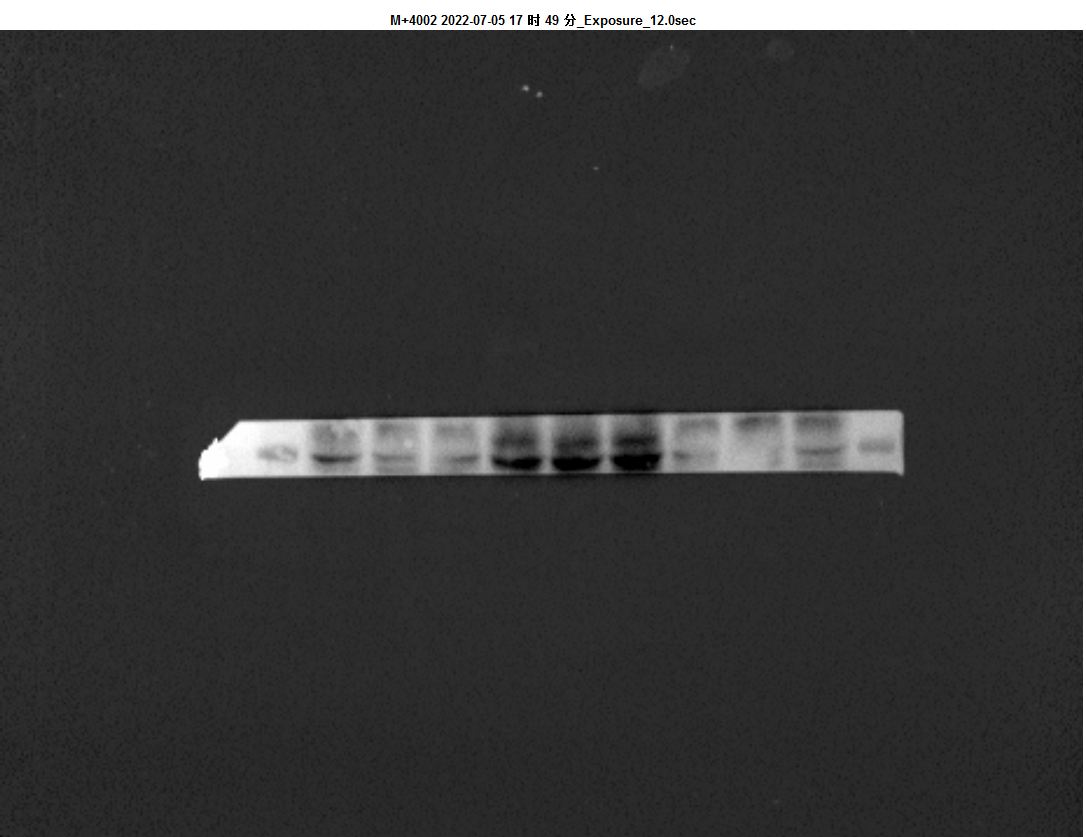


PINK1


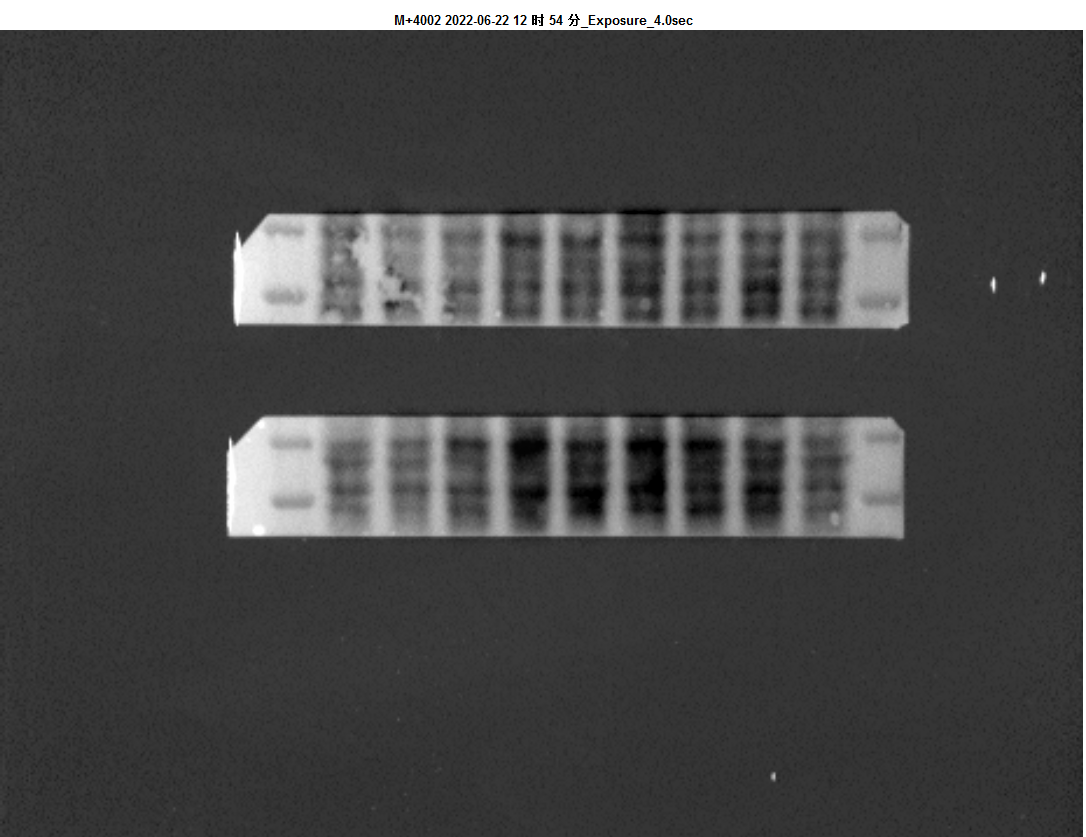


COXⅣ


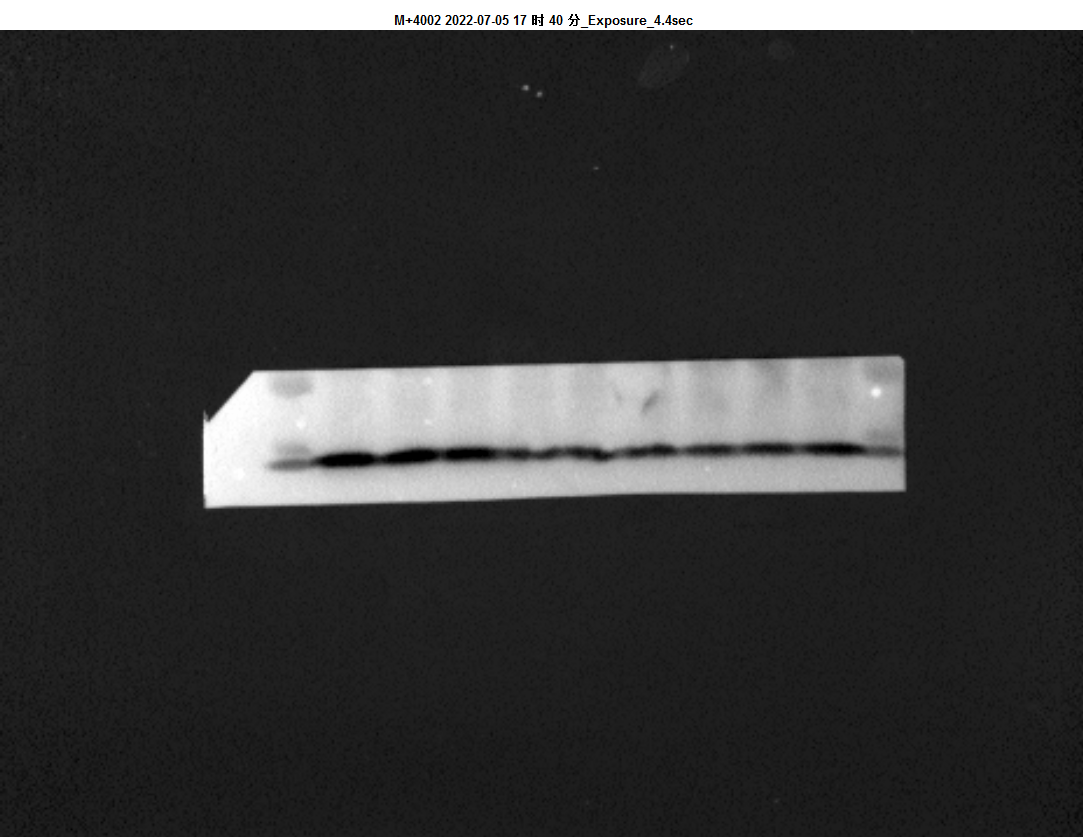


TLR4


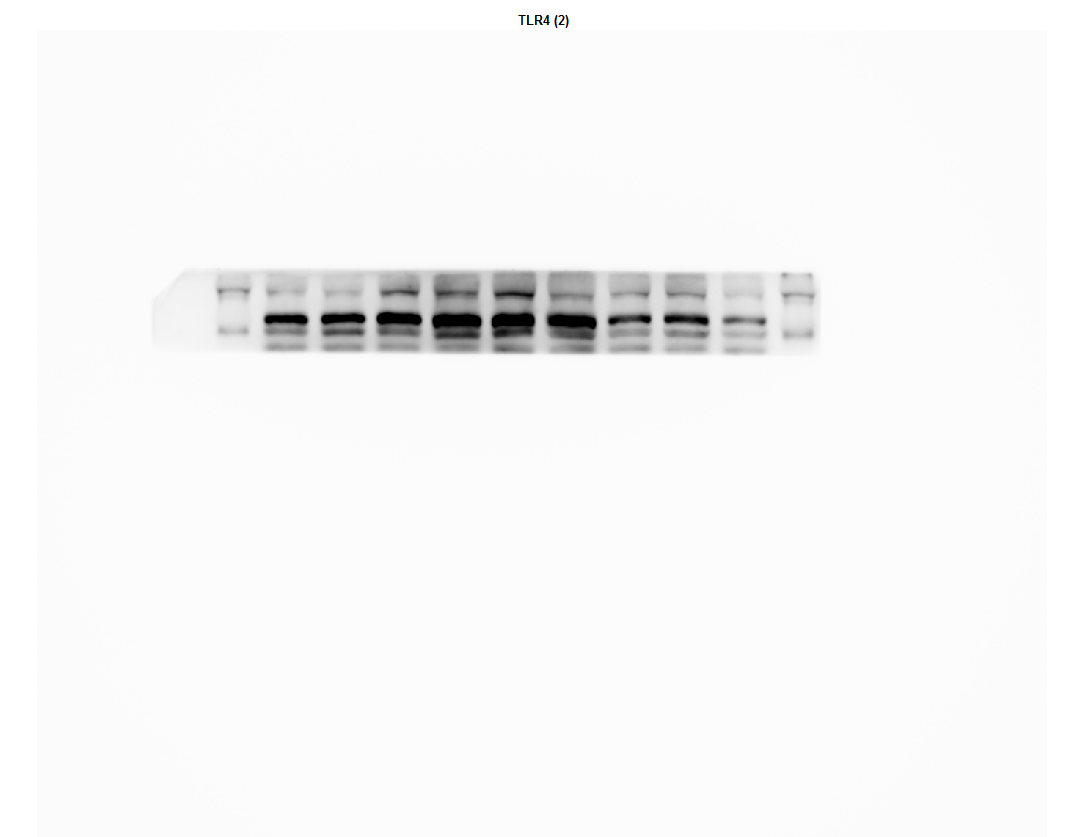


1. IKB


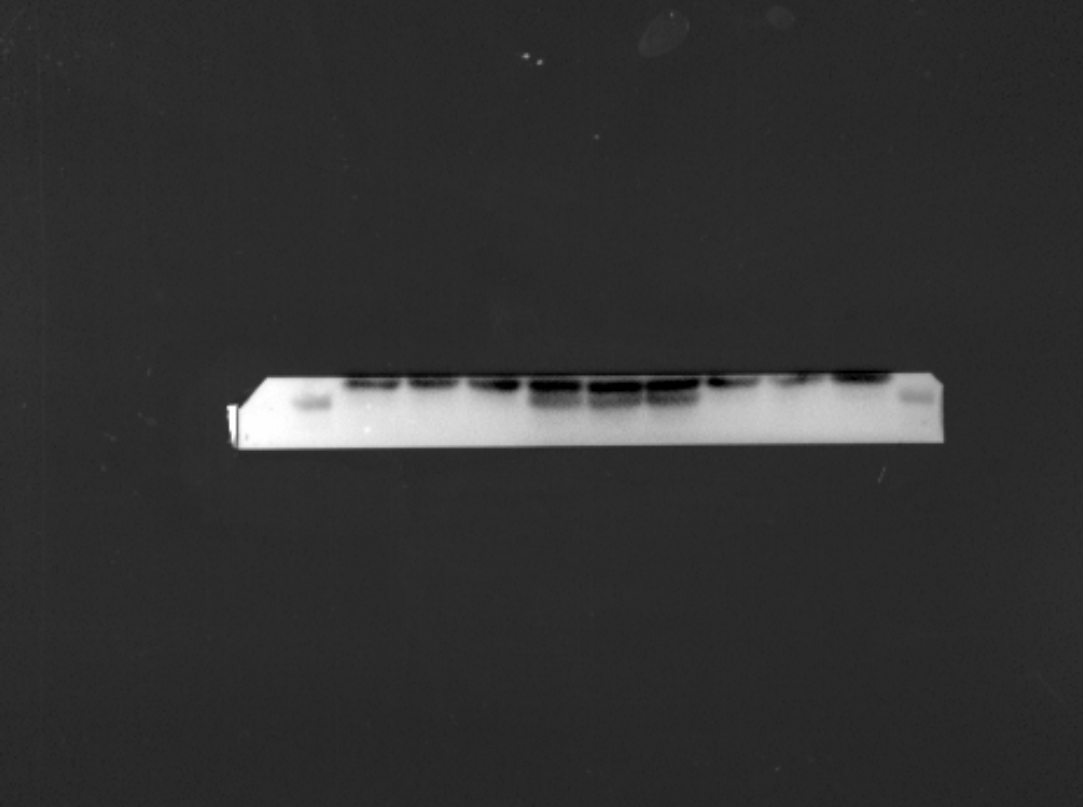


IKB


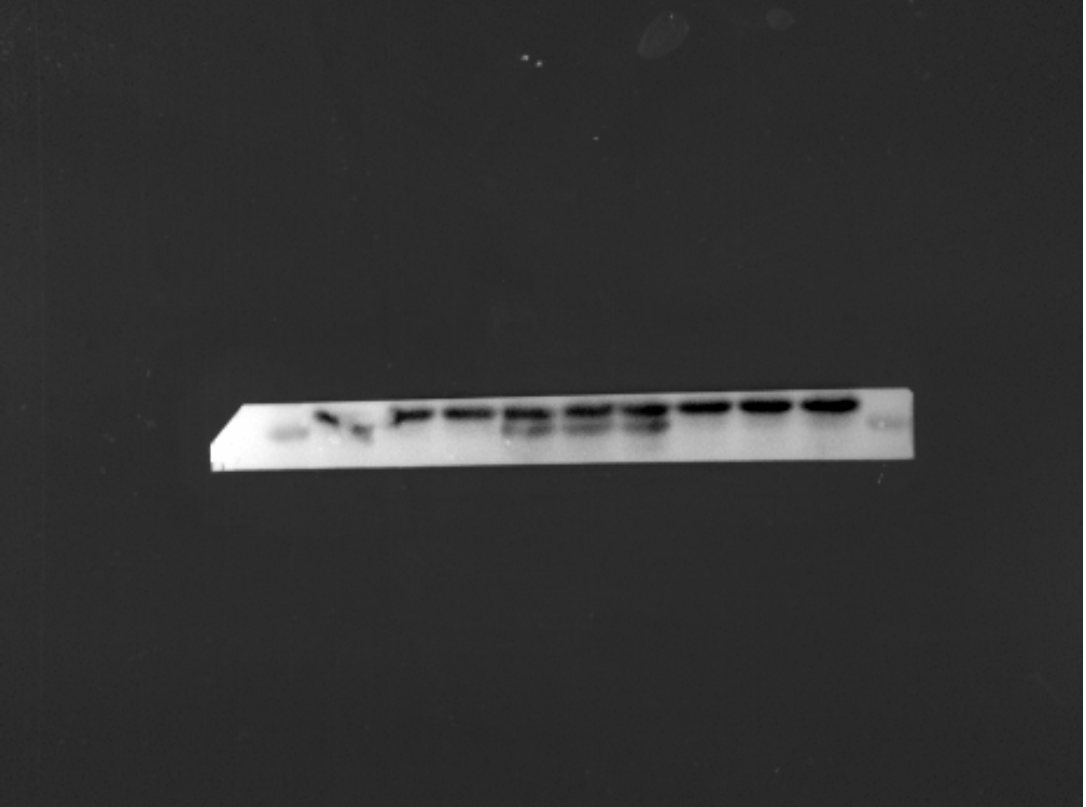


Nucle p65


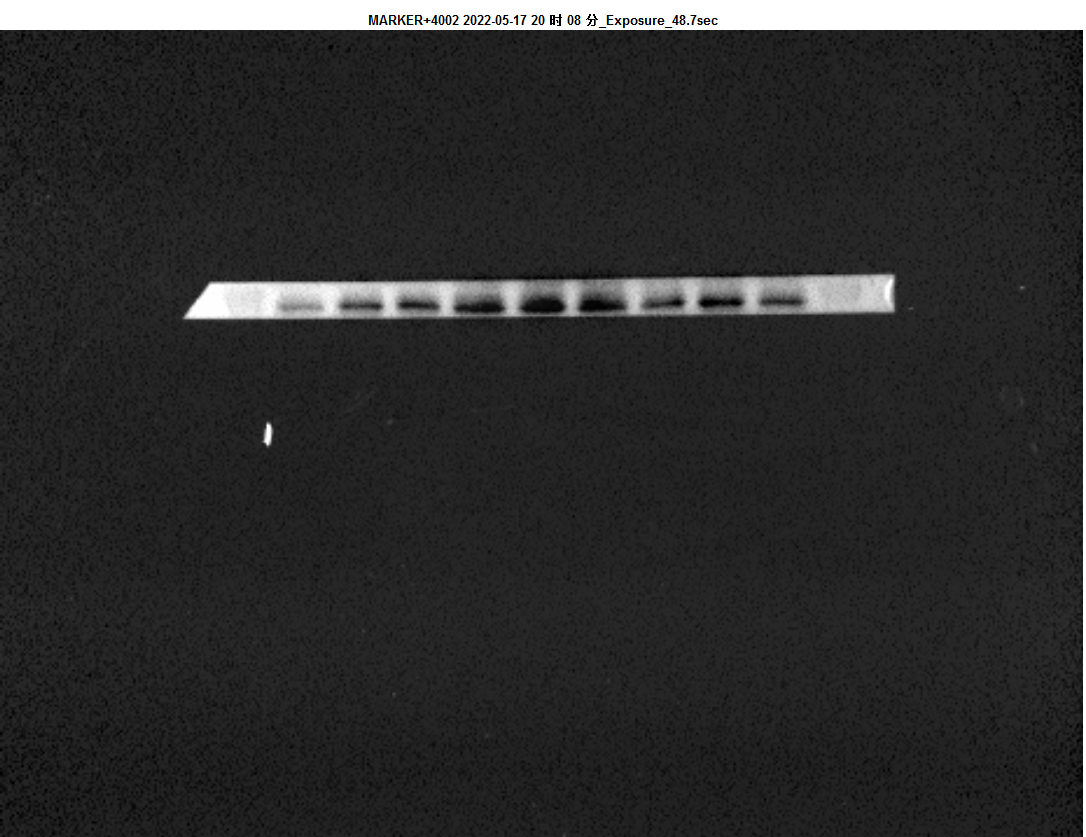


FOXA2


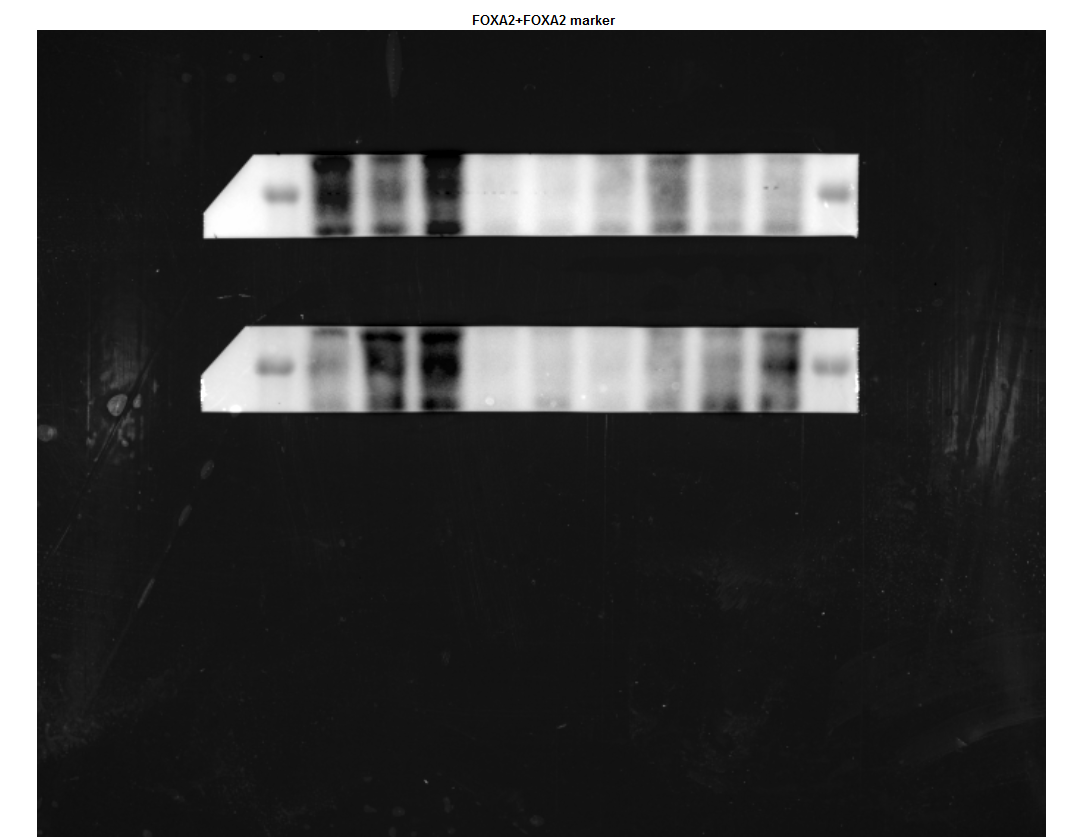


H3


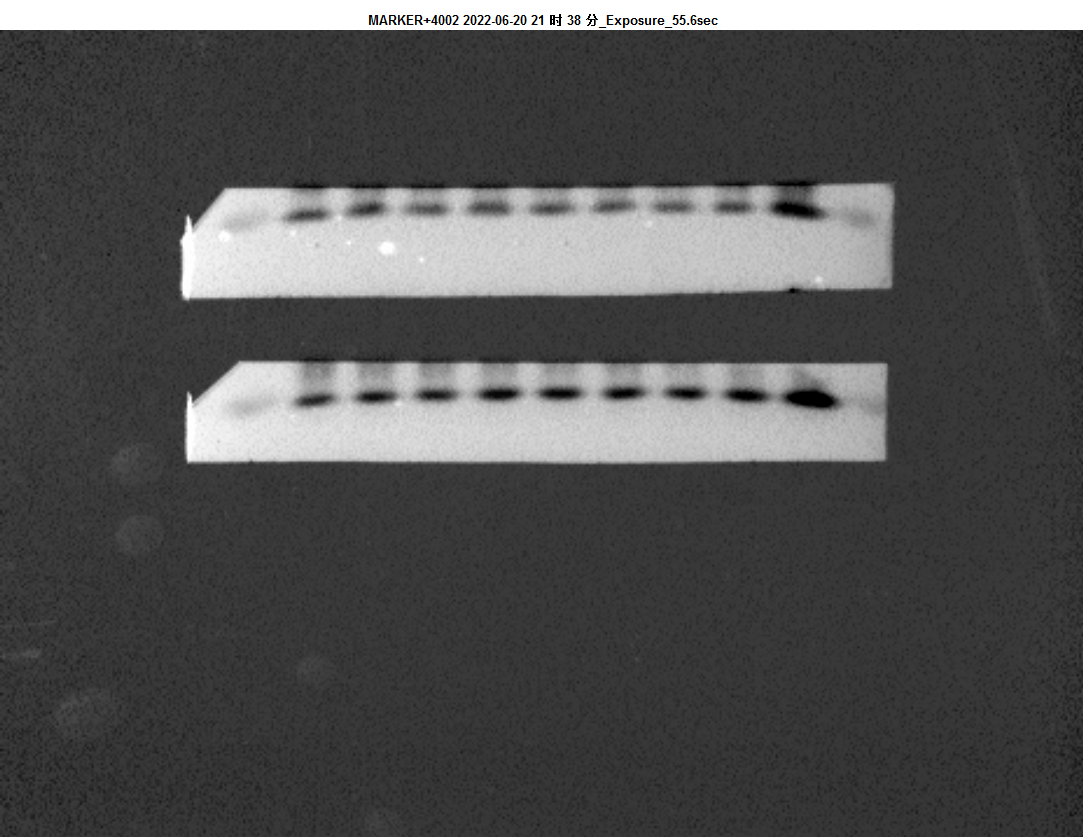


Cyto p65


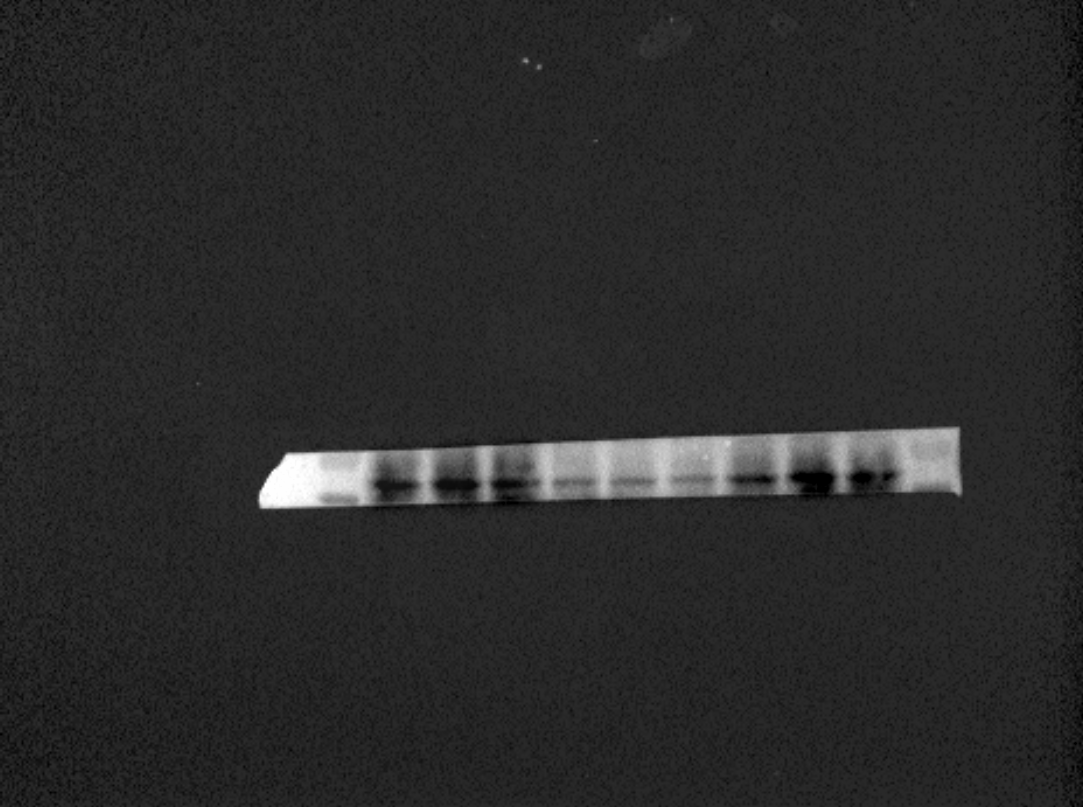


ACTB


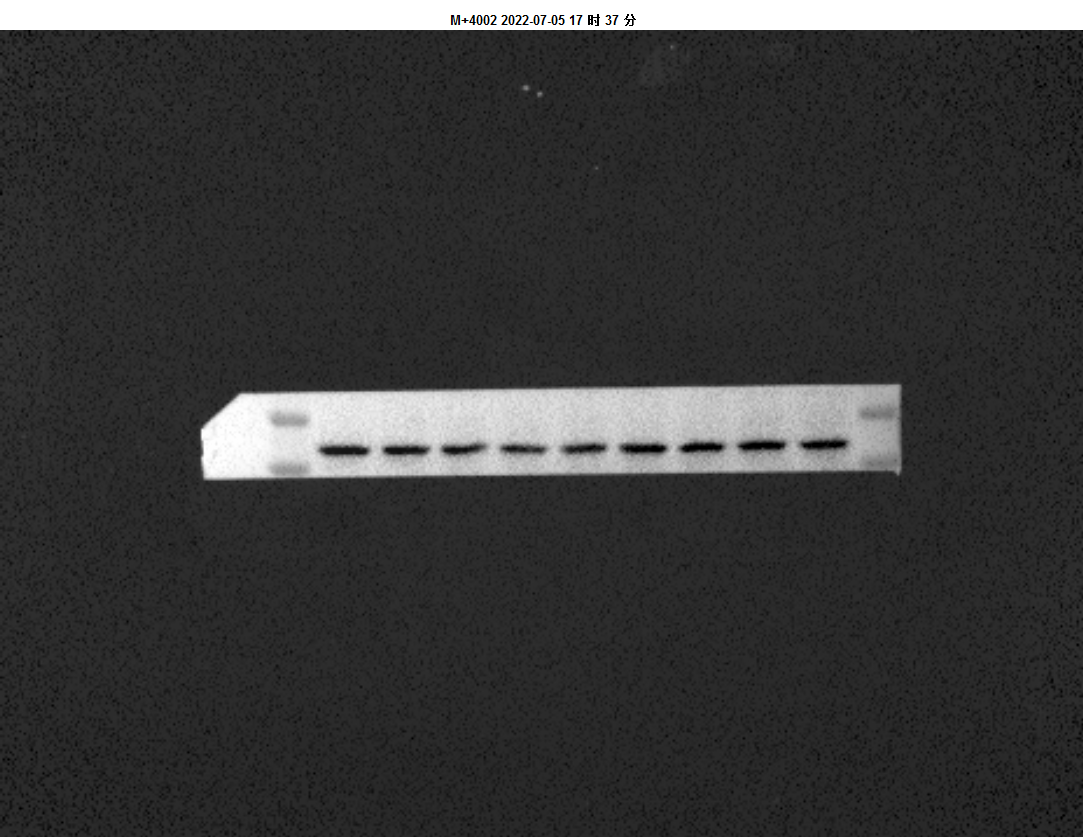

Supplement: Supplementary file 1 [file DataSheet_1.docx]
